# Serum BDNF and pro-BDNF levels in alcohol use disorders according to depression status: An exploratory study of their evolution two months after withdrawal

**DOI:** 10.1016/j.heliyon.2024.e38940

**Published:** 2024-10-04

**Authors:** Thibaut Gellé, Théodore Vinais, Aurélie Lacroix, Brigitte Plansont, Philippe Nubukpo, Murielle Girard

**Affiliations:** a Inserm U1094, IRD UMR270, Univ. Limoges, CHU Limoges, EpiMaCT - Epidemiology of chronic diseases in tropical zone, Institute of Epidemiology and Tropical Neurology, OmegaHealth, Limoges, France; b Research and Innovation Unit, Esquirol Hospital, 87025, Limoges, France

**Keywords:** BDNF, Pro-BDNF, Alcohol use disorders, Depression, Abstinence

## Abstract

**Background:**

Alcohol use disorders (AUDs) are complex pathologies with a myriad of molecular actors involved in both disease progression and remission. Brain-derived neurotrophic factor (BDNF) is suspected to be one such actor due to its neurotrophic effects. The BDNF precursor, pro-BDNF, has different effects, as it mainly promotes neuronal apoptosis. Both forms also play a role in depression and depressive episodes (DE). The aim of this exploratory study was to compare serum BDNF and pro-BDNF levels in patients with AUDs after withdrawal and according to DE status with those of controls without AUDs or DE.

**Materials and methods:**

Ninety-nine AUD patients and 40 controls were included. Questionnaires were used to assess both alcohol and psychiatric domains: the severity of hazardous alcohol consumption was assessed using Alcohol Use Disorders Identification Test (AUDIT), craving was assessed using Obsessive and Compulsive Drinking Scale (OCDS), anxiety was assessed with Hamilton Anxiety Rating Scale (HAM-A) and depression with Montgomery-Åsberg Depression Rating Scale (MADRS). Blood samples were collected during two visits: at the time of alcohol withdrawal (M0) and two months later (M2). ELISAs to measure serum BDNF and pro-BDNF levels were performed. AUD patients were categorized according to depression status at M2. Forty-five patients remained abstinent whereas 54 relapsed. BDNF serum levels rose after alcohol withdrawal, but pro-BDNF levels did not vary between M0 and M2.

**Results:**

AUD subjects without DE at M2 had higher BDNF levels at both M0 and M2 than AUD subjects with DE at M2. AUD subjects showed lower MADRS and OCD scores at M2 than at M0. AUD subjects without DE had lower BDNF levels at M0 than controls but not at M2, regardless of abstinence maintenance.

**Conclusion:**

BDNF serum levels were reduced in AUD patients compared to controls and were further reduced in patients with both AUDs and DE. Alcohol withdrawal treatment was sufficient to induce an increase in serum BDNF levels after 2 months, regardless of whether abstinence was maintained during this time period.

## Highlights

• Alcohol Use Disorders patients exhibit lower BDNF level in serum than controls.
• Alcohol withdrawal increase BDNF levels in AUD even if abstinence is not maintained.
• BDNF increase during alcohol withdrawal is not linked to a pro-BDNF decrease.
• Pro-BDNF may help select depressed AUD patients prone to recover after withdrawal.

## 1 Introduction

Alcohol use disorders (AUDs) and major depression (MD) account for half of the overall burden of disease attributable to mental and addictive disorders [1,2]. These two pathologies as well as depressive episode (DE) can be comorbidities of each other, as an example MD occurs in up to 55 % of patients who suffer from AUD as their primary pathology. However, the chronology of onset of those disorders remains unclear and varies among studies [3]. Their cause‒effect link is poorly known, although a specific symptomatology related to their comorbidity was observed [4]. Moreover, the direction of this possible cause-effect link remains difficult to determine as previous study have highlighted out that AUD could increase the risk of developing MD in a dose-effect association [5,6] and others have pointed out that MD could cause AUD through self-medication [7]. Sex appears to also play a role in the direction of such a link between the two pathologies showing a stronger association of MD causing AUD in females than in males [5].

In recent decades, the role of one pivotal molecule, brain-derived neurotrophic factor (BDNF), has received considerable attention, especially in the study of MD and DE. BDNF is an extracellular signaling protein known for its essential role in the development of the nervous system, including processes such as neurogenesis, neuroprotection and neurodegeneration [8]; synaptic plasticity [9]; and resistance to neuronal stress [10].

A recent meta-analysis showed a significant correlation between alcohol consumption and reduced BDNF levels in the blood [11]. Our team showed an increase in peripheral levels of BDNF after alcohol withdrawal [12], which may depend on the epigenetic regulation of the BDNF gene [13] and related biological characteristics of alcohol consumption [14]. Other results suggested that BDNF levels are related to concomitant liver changes [15]. BDNF levels are also diminished both in brain and throughout the body in patients during DE Those lower levels of BDNF are negatively correlated with the severity of the depressive symptoms [16].

BDNF is synthesized after proteolytic cleavage of its precursor, pro-BDNF [17]. Several studies have shown that pro-BDNF and BDNF have opposite effects [18,19]. BDNF binds mainly to tropomyosin-related kinase B receptors (TrkB) [20,21], which have anti-apoptotic properties and inhibit depression in the long term [22,23], whereas pro-BDNF preferentially binds to the p75^NTR^ receptor and promotes neuronal apoptosis and long-term depression [24].

The BDNF/pro-BDNF ratio at the peripheral and central levels is assumed to be a determinant of psychiatric disorders [25]. Our team reported an increase in the BDNF/pro-BDNF ratio during antidepressant treatment without any direct link to clinical improvement or depression severity, with patients exhibiting a "normalization" of BDNF levels approaching those of controls [26]. Similar BDNF/pro-BDNF ratios to those of controls were observed in alcohol-dependent individuals [27], but the evolution of this ratio after alcohol cessation and withdrawal treatment is not known.

In this exploratory study aiming to compare serum BDNF and pro-BDNF levels in patients with AUDs after withdrawal and according to DE status with those of controls without AUDs or DE, we measured both forms levels in the serum of AUD subjects with or without DE at the time of alcohol withdrawal and two months later. Our main hypothesis is that alcohol cessation in AUD patients is met with an increase in BDNF level in serum accompanied by a decrease in pro-BDNF level in serum. The presence of concurrent DE may be important for elucidating the involvement of these molecules in AUDs and DE. Consequently, the associations of serum levels of BDNF and pro-BDNF as well as their ratio with the outcome of subjects at two months after alcohol cessation and DE diagnosis were explored.

## 2 Materials and methods

### 2.1 Study population

Ninety-nine participants from an ongoing cohort study of alcohol-dependent individuals who were hospitalized to undergo alcohol detoxification in the psychiatric hospital of Limoges, France (Clinical Trial ID: NCT01491347), were included in this study within the first 48 h of hospitalization. The exclusion criteria were <18 years of age, potential of leaving the area in the first months after the beginning of the study, unstable somatic diseases, any suspected liver disease (e.g., according to routine examination and blood hepatic-enzyme levels), an unpredictable severe outcome (cancer), or acute somatic comorbidities (pancreatitis, hepatitis, etc.). The secondary exclusion criteria for this study were pregnancy and loss to follow-up at two months. The study received legal, administrative, and ethical approval from the French Committee for the Protection of Persons and the National Agency for Drug and Health Product Security (clinical trial ID: NCT01491347). All included participants provided written informed consent. The study followed the principles of the Declaration of Helsinki.

The study was described to patients hospitalized for AUDs, as diagnosed by their referent psychiatrist, in the first 48 h of alcohol withdrawal treatment.

Data from healthy controls (n = 40) were extracted from a previous publication by our team [26]. They were recruited from a group of blood donors not receiving antidepressant treatments and without AUDs as stated by the physician selecting the subjects for blood donation.

### 2.2 Data collection

Clinical and para-clinical evaluations were performed at inclusion (M0), which corresponded to the start of alcohol withdrawal, and at the two-month (M2) follow-up.

Clinical evaluation at inclusion was performed by a senior psychiatrist specialized in AUDs. Data were collected on age, sex, total alcohol consumption during the previous two months (M − 2 and M − 1) (converted into standard drinks per day) based on the results of the Alcohol Timeline Follow-Back (TLFB) [28,29] and tobacco use. Prescription of psychotropic treatments (antipsychotics, antidepressants, anxiolytics, mood stabilizers) was collected.

To assess alcohol consumption-related problems, the Alcohol Use Disorders Identification Test (AUDIT) was used. The AUDIT is a 10-item questionnaire that is used to screen for hazardous drinking even if alcohol-related problems have yet to manifest.

The severity of depression was measured using the 10-item Montgomery-Åsberg Depression Rating Scale (MADRS) [30,31]. The severity of anxiety symptoms was evaluated using the Hamilton Anxiety Rating Scale (HAM-A) with 14 items [[32], [33], [34]], and craving was evaluated by using the Obsessive and Compulsive Drinking Scale (OCDS), which is a 14-item questionnaire regarding obsessive and compulsive drinking ideas [35,36].

Participants were considered to be abstinent at M2 if they declared that they had not consumed alcohol during the previous two months. They were considered nonabstainers if they declared that they had consumed at least one standard drink since M0 according to the TLFB.

Participants were considered to have DE if diagnosed by a psychiatrist using DSM-V criteria (American Psychiatric Association, 2013).

### 2.3 Biological assessments

Fasting blood was collected at the time of inclusion (M0) and during the follow-up visit two months later (M2). Blood (6 mL) was collected in BD Vacutainer SST II Advance (ref 367955) and stored at 4 °C for up to 2 h before centrifugation for 5 min at 2300g and 4 °C. Then a portion of the serum was stored at −80 °C for future measurements. Measurements were made in 96-well plates using ELISA kits of free BDNF (dilution 1/50) (SK00752-01 Aviscera Bioscience) and pro-BDNF (dilution1/4) (SK00752-09 Aviscera Bioscience) according to the manufacturer's instructions (Aviscera Bioscience, Santa Clara, California, United States). The BDNF kit has a standard range of 3.9–250 pg/ml, a sensitivity of 1 pg/ml, an intra-CV: 4–6%, an inter-CV: 4–9% while the pro-BDNF kit has standard range of 62.5–4000 pg/ml, a sensitivity of 15 pg/ml, an intra-CV: 4–6% and an inter-CV: 4–8%. The BDNF/pro-BDNF ratio was then calculated based on the two serum concentrations.

The data extracted from Gelle et al. (2021) on MD subjects without AUDs and controls were used for comparison. BDNF and pro-BDNF levels were measured by the same operator using the same commercial kits and in the same time frame (6 months), ensuring the comparability of the data from these different studies. For controls, a single collection occurred, as their neurotrophin levels were considered stable over time and valid for comparison with AUD subjects at both M0 and M2.

### 2.4 Statistical analysis

Quantitative variables are described using medians [quartile 1, quartile 3], and comparisons between groups were carried out using the Mann‒Whitney *U* test due to the nonnormal distribution of the variables or the Kruskal‒Wallis test when comparing more than 3 groups. Normality of the data was assessed using the Kolmogorov-Smirnov test. The paired sample Wilcoxon test was used to compare data between M0 and M2 within the same subjects. Qualitative variables are described using frequencies and percentages, and group comparisons were conducted with the chi-square test.

Statistical analyses were performed using SPSS software (Version 27.0, Armonk, NY: IBM Corp), and p < 0.05 was considered to indicate statistically significant differences. Bonferroni's correction was used when performing multiple tests.

## 3 Results

### 3.1 Description of the study population

Ninety-nine participants (79 males and 20 females) were included and completed the follow-up two months after alcohol withdrawal. Their characteristics are summarized in Table 1. This population consisted of chronic alcohol users, with numerous previous withdrawals attempts (5.96 ± 7.40), heavy alcohol consumption during the 2 months prior to the study, and a vast majority of smokers (82.82 %). The frequency of major depression was reduced after withdrawal treatment (p < 0.001), dropping to 24.24 % at M2 from 65.65 % at M0. Anxiety was also relatively high at M0 and significantly decreased in the whole population at M2 (p < 0.001), as did OCDS scores and subscores (p < 0.001), indicating an improvement in the overall state of the subjects 2 months after alcohol withdrawal.

**Table 1:** Characteristics of AUDs population at M0 and M2 (n = 99).

		p value (comparison between M0 and M2)
Age (years)	48.00 [39.50; 53.50]	–
Sex ratio (n Male/n Female)	3.95 (79/20)	–
Tobacco use	82 (82.82 %)	–
Absence of DE at both M0 and M2	33 (33.33 %)	–
Current DE at M0 only	42 (42.42 %)	–
Current DE at M2 only	1 (1.01 %)	–
Current DE at both M0 and M2	23 (23.23 %)	–
Score MADRS M0	21.00 [7.50; 25.00]	–
Score MADRS M2	0.00 [0.00; 2.00]	<0.001
Score HAM-A M0	13.00 [9.00; 16.50]	–
Score HAM-A M2	5.00 [2.00; 8.00]	<0.001
Obsessive ideas M0	2.00 [1.00; 3.00]	–
Obsessive ideas M2	1.00 [0.00; 2.00]	<0.001
Compulsive ideas M0	9.00 [7.00; 10.00]	–
Compulsive ideas M2	1.00 [0.00; 4.50]	<0.001
Score OCDS total M0	11.00 [9.00; 13.00]	–
Score OCDS total M2	2.00 [1.00; 5.00]	<0.001
M0 Antidepressant only	25 (25.25 %)	–
M0 Neuroleptic only	37 (37.37 %)	–
M0 Both antidepressant and neuroleptic	26 (26.26 %)	–
Score AUDIT	30.00 [24.00; 34.00]	–
Days of alcohol consumption in the two months preceding hospitalization	57.00 [35.50; 60.00]	–
Average alcohol consumption per day in the two months preceding hospitalization (standard glass)	12.00 [7.50; 16.00]	–
BDNF M0	26.23 [19.56; 35.14]	–
BDNF M2	28.77 [23.42; 41.70]	<0.001
Pro-BDNF M0	7.69 [3.89; 15.11]	–
Pro-BDNF M2	8.83 [4.43; 18.11]	0.117
Ratio BDNF/Pro-BDNF M0	2.62 [1.55; 4.95]	–
Ratio BDNF/Pro-BDNF M2	3.15 [1.62; 5.20]	<0.001

Data are presented as n (%) or median [Q1; Q3].

Neurotrophin concentrations values are expressed in ng/mL.

BDNF levels significantly increased from M0 to M2 (p < 0.001), and the BDNF/pro-BDNF ratio also increased significantly (p < 0.001), while the pro-BDNF levels did not vary (p = 0.117).

### 3.2 Comparison of abstainers and nonabstainers at the time of alcohol withdrawal and 2 months after

Forty-five patients maintained abstinence, while 54 relapsed. Those groups, referred to as abstainers and nonabstainers, did not differ according to the sex ratio, age, tobacco use, pharmaceutical treatment and depression severity (assessed through MADRS scores). Although non-abstainers relapsed, they consumed less alcohol during the 2 months after withdrawal than in the 2 months prior to withdrawal. Both groups had significantly lower MADRS and OCDS scores and subscores at M2 than at M0 (p < 0.001). OCDS scores and subscores at M2 were significantly different between the two groups; although they decreased in both groups, this decrease was larger among abstainers (see Table 2). In the abstainer group, 15.55 % (7 out of 45) of patients had a depressive episode at M2, whereas 31.48 % (17 out of 54) of non-abstainers had DE at M2. Both groups had increased BDNF levels at M2 compared to M0 (p < 0.01 for abstainers and p < 0.001 for non-abstainers). Finally, the two groups differed in pro-BDNF levels at M0 but not at M2.

**Table 2:** Comparison between abstainers and non-abstainers at M2.

	Abstainers (n = 45)	Non-abstainers (n = 54)	p-value
Age (years)	50.00 [43.00; 56.00]	47.00 [39.00; 51.75]	0.079
Sex ratio (n Male/n Female)	4.00 (36/9)	3.95 (43/11)	1.000
Tobacco use M0	34 (75.56 %)	48 (88.89 %)	0.800
Tobacco use M2	35 (77.78 %)	49 (90.74 %)	0.730
MADRS M0	22.00 [6.00; 24.00]	20.00 [10.25; 26.00]	0.408
MADRS M2	0.00 [0.00; 1.00]	0.00 [0.00; 2.00]	0.297
Obsessive ideas M0	2.00 [1.00; 3.00]	2.00 [1.00; 3.00]	0.382
Obsessive ideas M2	0.00 [0.00; 1.00]	1.00 [0.25; 1.00]	0.003
Compulsive ideas M0	9.00 [7.00; 10.00]	9.00 [7.00; 11.00]	0.375
Compulsive ideas M2	0.00 [0.00; 0.00]	4.00 [2.00; 7.00]	<0.001
OCDS total M0	10.50 [9.00; 12.00]	11.00 [9.00; 13.00]	0.338
OCDS total M2	1.00 [0.00; 1.00]	4.50 [3.00; 8.75]	<0.001
M0 Antidepressant only	29 (64.44 %)	39 (72.22 %)	0.406
M0 Neuroleptic only	27 (60 %)	35 (64.81 %)	0.622
AUDIT M0	30.00 [24.00; 34.00]	31.00 [25.00; 34.00]	0.617
Days of alcohol consumption between M0 and M2	0.00 [0.00; 0.00]	10.50 [2.00; 22.75]	<0.001
Average alcohol consumption per day between M0 and M2	0.00 [0.00; 0.00]	7.00 [3.00; 11.75]	<0.001
Total alcohol consumed between M0 and M2	0.00 [0.00; 0.00]	53.50 [7.50; 186.50]	<0.001
BDNF M0	26.81 [20.10; 38.44]	24.93 [18.44; 32.72]	0.312
BDNF M2	28.77 [24.93; 41.35]	29.27 [22.46; 41.71]	0.768
Pro-BDNF M0	10.02 [5.14; 20.56]	6.29 [2.91; 11.11]	0.022
Pro-BDNF M2	10.68 [5.07; 23.88]	7.44 [2.44; 16.20]	0.114
Ratio BDNF/Pro-BDNF M0	2.29 [1.53; 3.34]	3.36 [1.63; 6.97]	0.085
Ratio BDNF/Pro-BDNF M2	3.09 [1.46; 5.16]	3.24 [1.79; 5.17]	0.374

Data are presented as n (%) or median [Q1; Q3].

Neurotrophin concentrations values are expressed in ng/mL.

### 3.3 Comparison between AUD subgroups according to DE status at M2

To assess variations in neurotrophin regarding DE status at M2, two subgroups of the AUD group were analyzed. The first subgroup contained AUD subjects without DE at M2, while the second contained AUD subjects with DE at M2. This sorting method did not take into account the DE status at M0. Subjects without DE at M2 had higher BDNF levels at both M0 and M2 than subjects with DE at M2 (Table 3). Moreover, subjects without DE at M2 had higher pro-BDNF levels at M2 than subjects with DE.

**Table 3:** Serum neurotrophin levels and psychometric tests comparison between AUDs groups according to DE status at M2.

	AUDs Without DE at M2 (n = 75)	AUDs With DE at M2 (n = 24)	p-value
MADRS M0	20.00 [6.00; 24.00]	23.00 [16.00; 27.75]	0.009
MADRS M2	4.00 [2.00; 7.00]	10.50 [7.00; 15.75]	<0.001
Obsessive ideas M0	2.00 [1.00; 3.00]	2.50 [1.25; 3; 00]	0.153
Obsessive ideas M2	1.00 [0.00; 1.00]	1.00 [1.00; 2.00]	<0.001
Compulsive ideas M0	9.00 [7.00; 10.00]	10.00 [8.00; 11.00]	0.038
Compulsive ideas M2	1.00 [0.00; 3.00]	5.00 [1.25; 8.00]	<0.001
OCDS total M0	10.50 [8.00; 13.00]	12.00 [10.00; 14.00]	0.044
OCDS total M2	1.00 [0.00; 3.00]	6.00 [3.00; 9.75]	<0.001
BDNF M0	27.15 [21.25; 36.22]	21.90 [16.64; 27.27]	0.033
BDNF M2	29.65 [25.32; 41.81]	22.71 [20.42; 33.09]	0.011
Pro-BDNF M0	9.03 [4.06; 16.59]	5.88 [3.33; 7.70]	0.074
Pro-BDNF M2	10.82 [4.88; 22.00]	5.00 [0.40; 8.80]	0.006
Ratio BDNF/Pro-BDNF M0	2.56 [1.49; 4.51]	3.45 [1.72; 7.00]	0.242
Ratio BDNF/Pro-BDNF M2	3.02 [1.50; 5.06]	4.08 [2.68; 6.12]	0.084

Data are presented as median [Q1; Q3].

Neurotrophin concentrations values are expressed in ng/mL.

No difference between the two DE subgroups of AUD patients at M2 was observed in the BDNF/pro-BDNF ratio. When comparing between M0 and M2 in a same subgroup, AUD patients without DE at M2 showed higher BDNF at M2 (p < 0.05) as well as an increase of BDNF/Pro-BDNF ratio (p < 0.05). For AUD patients with DE at M2, an increase of BDNF at M2 was detected (p < 0.001).

### 3.4 Comparison of controls with abstainers and nonabstainers, both adjusted for absence of DE at M2

No significant differences were identified between the two AUD subgroups (abstainers and nonabstainers), as presented in Table 4. When comparing them to controls, similar results were obtained because both AUD subgroups showed significantly lower levels of BDNF than the control group (p < 0.001 for abstainers without DE at M2 and p < 0.01 for non-abstainers without DE at M2) as well as a lower BDNF/pro-BDNF ratio (p < 0.001 for both subgroups) at M0. No difference was observed between AUD subgroups and controls in pro-BDNF levels at M0 or M2. No further difference was observed across any forms of this neurotrophin at M2.

**Table 4:** Serum neurotrophin levels comparison between abstainers and non-abstainers, both without DE at M2, and controls.

	Abstainers without DE at M2 (n = 38)	Non abstainers without DE at M2 (n = 37)	Controls (n = 40)
BDNF M0	28.43 [21.40; 39.94]	26.71 [20.28; 32.87]	37.93 [37.63; 39.17]
BDNF M2	28.96 [25.56; 41.76]	30.93 [23.98; 41.72]	
Pro-BDNF M0	11.04 [4.79; 21.93]	7.87 [3.64; 11.59]	9.01 [7.34; 9.78]
Pro-BDNF M2	11.20 [5.05; 25.05]	9.60 [4.71; 19.64]	
Ratio BDNF/Pro-BDNF M0	2.25 [1.46; 3.22]	2.97 [1.54; 5.80]	4.30 [3.87; 5.47]
Ratio BDNF/Pro-BDNF M2	3.28 [1.44; 5.32]	2.84 [1.63; 4.27]	

Data are presented as median [Q1; Q3].

Neurotrophin concentrations values are expressed in ng/mL.

## 4 Discussion

In this exploratory study, we measured BDNF and pro-BDNF levels in the serum of AUD subjects at the time of alcohol withdrawal treatment and two months later, and we showed that cessation of alcohol was followed by an increase in BDNF levels. We also showed that this increase in BDNF levels was not linked to a reduction in pro-BDNF levels. Another important finding of this work was that AUD subjects without DE after withdrawal had consistently higher BDNF levels than AUD subjects with DE after withdrawal.

### 4.1 Neurotrophin variations after alcohol withdrawal and at follow-up

#### 4.1.1 A. neurotrophin variations in the entire AUD sample

We found an increase in both BDNF levels and the BDNF/pro-BDNF ratio after alcohol withdrawal. This suggests a shift in the BDNF/pro-BDNF balance in favor of BDNF both in serum and in the brain as variation in blood levels of BDNF are suggested to take place at the same time in the brain [37]. However, peripheral BDNF may not originate from the brain as it is produced by other organs such as skin, lung, heart or skeletal muscle [38] and can be stored by platelets. In the case of platelets, BDNF's origin remains unknown [39]. This increase in BDNF levels after withdrawal is in accordance with a previous study by Ornell and colleagues. They hypothesized that the early increase detected after withdrawal was more likely a direct effect of withdrawal rather than intoxication-related substance effects [40]. The increase observed here two months after withdrawal could be the continuation of this early increase. Interestingly, although BDNF levels increased, the pro-BDNF levels did not decrease, as we otherwise would have expected from its conversion into BDNF and BDNF propeptide [25]. Another explanation is that pro-BDNF acts through a different pathway than the one classically described involving p75NTR/sortilin and damages neurons, culminating in neuronal apoptosis [41]. A further study of both BDNF propeptide and BDNF mRNA after alcohol withdrawal and 2 months after alcohol withdrawal could further elucidate how BDNF levels increase while pro-BDNF levels remain steady.

#### 4.1.2 B. neurotrophin differences between abstainers and nonabstainers

When comparing neurotrophin level changes between the two AUD subgroups, we observed an increase in BDNF levels in both subgroups after alcohol withdrawal. This finding bolsters the idea that alcohol withdrawal leads to an increase in BDNF levels. It is important to note that the increase of BDNF levels in both subgroups reflects not only alcohol cessation but also that both groups diminished their alcohol consumption during the two months follow-up. The initial withdrawal period at M0 and the reduction of consumption that followed can both be involved in the increase of BDNF level observed in this study. BDNF's neuroplasticity-inducing properties and capacity to promote cell survival in the central nervous system [9,10] could be useful for mitigating the negative impact on neurogenesis of prolonged and regular consumption of alcohol [42,43]. Pro-BDNF levels and the BDNF/pro-BDNF ratio did not change between the initiation of alcohol withdrawal and the follow-up visit two months later in either of those subsamples. However, direct comparison revealed a significantly higher level of pro-BDNF at M0 in the abstainer subsample. Since this variation in neurotrophin was the only difference observed, it suggests that higher pro-BDNF levels might predict the ability to maintain abstinence. This possible role would not require pro-BDNF maturation into BDNF, as BDNF levels were similar at M2 between abstainers and nonabstainers. This finding further reinforces the idea of a different pathway for pro-BDNF than p75NTR/sortilin. Pro-BDNF is also known to bind to Trk-B to induce phosphorylation and participate in the ERK1/2 signaling pathway [[44], [45], [46]]. Fayard and colleagues have shown that this pathway promotes neurite outgrowth in PC12-TrkB cells, demonstrating that pro-BDNF does not solely lead to neuronal apoptosis but could also promote cell survival under specific conditions.

#### 4.1.3 C. neurotrophin differences according to depressive status at M2

The new subsamples took into account the depressive status at M2 but not at M0. This is in order to alleviate potential bias from depressive symptoms induced solely by alcohol consumption [5]. This could be the case of up to 42 patients who were diagnosed with DE at M0 but not at M2. We found that the subsample without DE at M2 had higher BDNF levels at both M0 and M2 than the subsample with DE at M2. The lower BDNF levels of depressed AUD patients after alcohol withdrawal are contrary to the findings of a previous study by Han and colleagues that reported no significant differences in BDNF levels between samples of AUD patients with and without depression [47]. These inconsistent results could be explained by differences in the time points of BDNF measurements. In our study, blood was drawn the morning after admission to the hospital, which corresponded to the cessation of alcohol consumption, whereas Han and colleagues drew blood from patients who were prohibited from drinking alcohol for at least one week prior. It was previously suggested that BDNF levels tend to decrease in the early stages of abstinence due to the long latency needed for BDNF levels to increase [48]. Therefore, the aforementioned study might have measured BDNF levels in this long latency period. The higher BDNF levels at M0 in patients without DE at M2 could also indicate a difference between good and bad responders to alcohol withdrawal that translates to depression status. A higher baseline BDNF level might facilitate recovery from a possible depressive episode at the time of alcohol withdrawal and maintenance of a steady mental state for a short period afterward (2 months, in our case). Pro-BDNF levels at M2 were also higher in the subsample without DE at M2, which is unexpected, as previous studies have shown that pro-BDNF levels are higher in patients suffering from depressive episodes than in controls [26,49,50]. No variations in pro-BDNF levels within the same sample were detected between M0 and M2, which could indicate that the difference between the two subsamples at M2 might be due to subtle changes in neurotrophin production that arose from alcohol withdrawal. The ratio between both forms increased between M0 and M2 in the subsample without DE, meaning that the increase in BDNF levels was enough to disrupt the balance. The BDNF/pro-BDNF ratio is known to be dysregulated in AUD patients [41], and our results suggest further dysregulation induced by depressive episodes in an AUD subsample.

#### 4.1.4 D. neurotrophin differences according to abstinence status and depressive status at M2 from a control group

. First, it is important to note that we did not find any differences in neurotrophin levels between the two AUD subgroups. Few patients (15.55 %,7 out of 45) in the abstainer subsample had a depressive episode at M2, whereas 31.48 % (17 out of 54) of patients in the nonabstainer subsample had DE at M2. These results suggest that the difference in pro-BDNF levels that we observed previously between abstainers and nonabstainers might not be predictive of the maintenance of abstinence but rather of the proportion of individuals with depressive episodes at M2 in the two samples, as pro-BDNF levels increase during depression [26,50]. Comparison of these subgroups to the control group, revealed that BDNF levels were lower in all AUD patients. This is in accordance with previous findings of our team as well as the literature [25,26,50]. This difference in BDNF levels translates into a difference in the BDNF/pro-BDNF ratio between both AUD subgroups and the control group, showing that this ratio is dysregulated in AUD patients regardless of DE status. Finally, we did not find any difference in pro-BDNF levels between the AUD subsamples and the controls. Thus, our results indicate that pro-BDNF levels alone might not distinguish between these subsamples.

### 4.2 Overall status after alcohol withdrawal and at follow-up

In this study, we were able to show that depressive episodes were less frequent among AUD patients 2 months after alcohol withdrawal than they were previously. Both AUDs and depression are associated with a depletion of serum BDNF levels, which might indicate a common pathway in their physiopathology. We also showed that anxiety (measured by the HAM-A), as well as both obsessive and compulsive drinking ideas (measured by the OCDS), were lower 2 months after alcohol withdrawal. Comparison between abstainers and non-abstainers showed a reduction in compulsive and obsessive drinking ideas, meaning that regardless of the maintenance of abstinence, the withdrawal period was successful because it helped patients reduce intrusive thoughts linked to alcohol cravings. Therefore, even if AUD patients relapse, they are more likely to reduce either the frequency of drinking or the quantity of alcohol consumed. However, this has to be put in perspective with the risks associated with alcohol withdrawal symptoms without the care of medical professionals. Indeed, repeated withdrawal attempts can be detrimental to brain health, as they may cause neurological complications such as epilepsy, delirium tremens and cognitive deficits [51].

While the OCDS total score was significantly lower in both subgroups at M2 compared to M0, it was significantly lower in the abstainers than in the nonabstainers. This is in accordance with the predictive value of the OCDS for the maintenance of abstinence demonstrated in the literature [52] and with our previous findings [15].

### 4.3 Limitations and strengths

In this study, we showed that changes in BDNF levels occurred directly after alcohol withdrawal and two months after. BDNF levels are influenced by both AUDs and DE although it remains unclear if the same pathways are used in both cases. This study has several limitations. First, due to the settings of this study, multiple factors that could influence peripheral BDNF levels could not be controlled such as alcohol consumption pattern at M0 or physical activity, prescribed medicine and platelet counts. Second, our measurement methods (relying on ELISA) did not allow us to study changes in neurotrophin levels in further detail. Changes in expression of the neurotrophin of interest at the mRNA level could have given more insight about subtle changes that might be undetectable with ELISA essay. Third, we did not collect measurements from controls at M2. Although changes in neurotrophin levels were not expected in the control group, conducting measurements at M2 in controls would have allowed us to better compare AUD subjects with controls. Another limitation resides in the sex ratio of our sample. With only 20 females in our sample, a potential sex effect could pass unnoticed and therefore should be investigated in future studies. Finally, physical activity was not taken into account in our study but could act as cofounding factor as it has been shown to increase BDNF levels in serum [53]. In contrast, the main strength of our study is the inclusion of multiple subsamples according to abstinence and depression status. This allowed us to better assess the effects of those disorders on BDNF levels, especially in individuals with concurrent AUDs and DE.

## 5 Conclusion

BDNF and its precursor play an important role in cerebral plasticity as well as neuronal survival and are strongly implicated in the physiopathology of AUDs, MD and DE. BDNF levels increase after alcohol cessation in AUD patients independently of maintenance of abstinence. The roles of BDNF and pro-BDNF in the maintenance of abstinence are unclear, as alcohol withdrawal, even when followed by relapse, can induce an increase in BDNF levels. However, measuring neurotrophin, especially pro-BDNF, at the time of cessation of alcohol could allow differentiation between good and bad responders in AUD patients with DE. Further studies are required to identify the pathways and molecular interactions through which BDNF and pro-BDNF participate in the physiopathology of AUDs MD and DE, as they seem to share molecular actors and could also share similar mechanisms.

## Funding

This work was supported by the 10.13039/501100009243French Health Ministry (Direction Générale de l’Offre de Soin) [grant number PHRC-2011: API-04-08].

## Ethical Statement

This work has been carried out in accordance with The Code of Ethics of the World Medical Association (Declaration of Helsinki) for experiments involving humans. The study received legal, administrative, and ethical authorizations from the French Committee for the Protection of Persons and the National Agency for Drug and Health Product Security (Clinical Trial ID: NCT01491347). All included participants provided their written informed consent.

## Data availability

Data will be made available on request. Data associated with this study has not been deposited into a publicly available repository.

## CRediT authorship contribution statement

**Thibaut Gellé:** Writing – review & editing, Methodology, Investigation. **Théodore Vinais:** Writing – original draft, Formal analysis. **Aurélie Lacroix:** Writing – original draft, Investigation. **Brigitte Plansont:** Investigation. **Philippe Nubukpo:** Writing – review & editing, Supervision, Funding acquisition, Conceptualization. **Murielle Girard:** Writing – review & editing, Methodology, Investigation, Funding acquisition, Formal analysis, Conceptualization.

## Declaration of competing interest

The authors declare the following financial interests/personal relationships which may be considered as potential competing interests:Philippe Nubukpo reports financial support was provided by French Ministry of Health and Access to Care. If there are other authors, they declare that they have no known competing financial interests or personal relationships that could have appeared to influence the work reported in this paper.

## References

[1] Degenhardt L., Charlson F., Ferrari A., Santomauro D., Erskine H., Mantilla-Herrara A., Whiteford H., Leung J., Naghavi M., Griswold M., Rehm J., Hall W., Sartorius B., Scott J., Vollset S.E., Knudsen A.K., Haro J.M., Patton G., Kopec J., Vos T. (2018). The global burden of disease attributable to alcohol and drug use in 195 countries and territories, 1990–2016 : a systematic analysis for the Global Burden of Disease Study 2016. Lancet Psychiatr..

[2] GBD 2019 Mental Disorders Collaborators (2022). Global, regional, and national burden of 12 mental disorders in 204 countries and territories, 1990-2019 : a systematic analysis for the Global Burden of Disease Study 2019. Lancet Psychiatr..

[3] Cranford J.A., Nolen-Hoeksema S., Zucker R.A. (2011). Alcohol involvement as a function of co-occurring alcohol use disorders and major depressive episode : evidence from the National Epidemiologic Survey on Alcohol and Related Conditions. Drug Alcohol Depend..

[4] Carton L., Pignon B., Baguet A., Benradia I., Roelandt J.-L., Vaiva G., Thomas P., Amad A., De Timary P., Naassila M., Geoffroy P.A., Rolland B. (2018). Influence of comorbid alcohol use disorders on the clinical patterns of major depressive disorder : a general population-based study. Drug Alcohol Depend..

[5] Boden J.M., Fergusson D.M. (2011). Alcohol and depression. Addiction.

[6] Lasserre A.M., Imtiaz S., Roerecke M., Heilig M., Probst C., Rehm J. (2022). Socioeconomic status, alcohol use disorders, and depression : a population-based study. J. Affect. Disord..

[7] Turner S., Mota N., Bolton J., Sareen J. (2018). Self‐medication with alcohol or drugs for mood and anxiety disorders : a narrative review of the epidemiological literature. Depress. Anxiety.

[8] Mattson M.P., Maudsley S., Martin B. (2004). BDNF and 5-HT : a dynamic duo in age-related neuronal plasticity and neurodegenerative disorders. Trends Neurosci..

[9] Murray P.S., Holmes P.V. (2011). An overview of brain-derived neurotrophic factor and implications for excitotoxic vulnerability in the hippocampus. International Journal of Peptides.

[10] Marosi K., Mattson M.P. (2014). BDNF mediates adaptive brain and body responses to energetic challenges. Trends Endocrinol. Metabol.: TEM (Trends Endocrinol. Metab.).

[11] Shafiee A., Jafarabady K., Rafiei M.A., Beiky M., Seighali N., Golpayegani G., Jalali M., Soltani Abhari F., Arabzadeh Bahri R., Safari O., Bakhtiyari M., Alirezaei A. (2023). Effect of alcohol on Brain-Derived Neurotrophic Factor (BDNF) blood levels : a systematic review and meta-analysis. Sci. Rep..

[12] Costa M.-A., Girard M., Dalmay F., Malauzat D. (2011). Brain-derived neurotrophic factor serum levels in alcohol-dependent subjects 6 months after alcohol withdrawal. Alcohol Clin. Exp. Res..

[13] Lacroix A., Ramoz N., Girard M., Plansont B., Poupon D., Gorwood P., Nubukpo P. (2023). BDNF CpG methylation and serum levels covary during alcohol withdrawal in patients with alcohol use disorder : a pilot study. World J. Biol. Psychiatr.: The Official Journal of the World Federation of Societies of Biological Psychiatry.

[14] Nubukpo P., Ramoz N., Girard M., Malauzat D., Gorwood P. (2017). Determinants of blood brain-derived neurotrophic factor blood levels in patients with alcohol use disorder. Alcohol Clin. Exp. Res..

[15] Girard M., Labrunie A., Malauzat D., Nubukpo P. (2020). Evolution of BDNF serum levels during the first six months after alcohol withdrawal. World J. Biol. Psychiatr.: The Official Journal of the World Federation of Societies of Biological Psychiatry.

[16] Cavaleri D., Moretti F., Bartoccetti A., Mauro S., Crocamo C., Carrà G., Bartoli F. (2023). The role of BDNF in major depressive disorder, related clinical features, and antidepressant treatment : insight from meta-analyses. Neurosci. Biobehav. Rev..

[17] Koshimizu H., Kiyosue K., Hara T., Hazama S., Suzuki S., Uegaki K., Nagappan G., Zaitsev E., Hirokawa T., Tatsu Y., Ogura A., Lu B., Kojima M. (2009). Multiple functions of precursor BDNF to CNS neurons : negative regulation of neurite growth, spine formation and cell survival. Mol. Brain.

[18] Binder D.K., Scharfman H.E. (2004). Brain-derived neurotrophic factor. Growth Factors.

[19] De Vincenti A.P., Ríos A.S., Paratcha G., Ledda F. (2019). Mechanisms that modulate and diversify BDNF functions : implications for hippocampal synaptic plasticity. Front. Cell. Neurosci..

[20] Klein R., Nanduri V., Jing S., Lamballe F., Tapley P., Bryant S., Cordon-Cardo C., Jones K.R., Reichardt L.F., Barbacid M. (1991). The trkB tyrosine protein kinase is a receptor for brain-derived neurotrophic factor and neurotrophin-3. Cell.

[21] Noble E.E., Billington C.J., Kotz C.M., Wang C. (2011). The lighter side of BDNF. Am. J. Physiol. Regul. Integr. Comp. Physiol..

[22] Pang P.T., Teng H.K., Zaitsev E., Woo N.T., Sakata K., Zhen S., Teng K.K., Yung W.-H., Hempstead B.L., Lu B. (2004). Cleavage of proBDNF by tPA/plasmin is essential for long-term hippocampal plasticity. Science.

[23] Teng H.K., Teng K.K., Lee R., Wright S., Tevar S., Almeida R.D., Kermani P., Torkin R., Chen Z.-Y., Lee F.S., Kraemer R.T., Nykjaer A., Hempstead B.L. (2005). ProBDNF induces neuronal apoptosis via activation of a receptor complex of p75NTR and sortilin. J. Neurosci.: The Official Journal of the Society for Neuroscience.

[24] Woo N.H., Teng H.K., Siao C.-J., Chiaruttini C., Pang P.T., Milner T.A., Hempstead B.L., Lu B. (2005). Activation of p75NTR by proBDNF facilitates hippocampal long-term depression. Nat. Neurosci..

[25] Yang B., Ren Q., Zhang J.-C., Chen Q.-X., Hashimoto K. (2017). Altered expression of BDNF, BDNF pro-peptide and their precursor proBDNF in brain and liver tissues from psychiatric disorders : rethinking the brain-liver axis. Transl. Psychiatry.

[26] Gelle T., Samey R.A., Plansont B., Bessette B., Jauberteau-Marchan M.-O., Lalloué F., Girard M. (2021). BDNF and pro-BDNF in serum and exosomes in major depression : evolution after antidepressant treatment. Progress in Neuro-Psychopharmacology & Biological Psychiatry.

[27] Zhou C., Zhong J., Zou B., Fang L., Chen J., Deng X., Zhang L., Zhao X., Qu Z., Lei Y., Lei T. (2017). Meta-analyses of comparative efficacy of antidepressant medications on peripheral BDNF concentration in patients with depression. PLoS One.

[28] Cervantes E.A., Miller W.R., Tonigan J.S. (1994). Comparison of timeline follow-back and averaging methods for quantifying alcohol consumption in treatment research. Assessment.

[29] Grant K.A., Tonigan J.S., Miller W.R. (1995). Comparison of three alcohol consumption measures : a concurrent validity study. J. Stud. Alcohol.

[30] Montgomery S.A., Asberg M. (1979). A new depression scale designed to be sensitive to change. Br. J. Psychiatr.: J. Ment. Sci..

[31] Peyre F., Martinez R., Calache M., Verdoux H., Bourgeois M. (1989). [New validation of the Montgomery and Asberg Depression Scale (MADRS) on a sample of 147 hospitalized depressed patients]. Ann. Med.-Psychol..

[32] Guelfi J.D. (1997).

[33] Guelfi J.D. (1996).

[34] Hamilton M. (1959). The assessment of anxiety states by rating. Br. J. Med. Psychol..

[35] Ansseau M., Besson J., Lejoyeux M., Pinto E., Landry U., Cornes M., Deckers F., Potgieter A., Ades J. (2000). A French translation of the obsessive-compulsive drinking scale for craving in alcohol-dependent patients : a validation study in Belgium, France, and Switzerland. Eur. Addiction Res..

[36] Anton R.F., Moak D.H., Latham P. (1995). The Obsessive Compulsive Drinking Scale : a self-rated instrument for the quantification of thoughts about alcohol and drinking behavior. Alcohol Clin. Exp. Res..

[37] Lee B.-H., Kim H., Park S.-H., Kim Y.-K. (2007). Decreased plasma BDNF level in depressive patients. J. Affect. Disord..

[38] Esvald E.-E., Tuvikene J., Kiir C.S., Avarlaid A., Tamberg L., Sirp A., Shubina A., Cabrera-Cabrera F., Pihlak A., Koppel I., Palm K., Timmusk T. (2023). Revisiting the expression of BDNF and its receptors in mammalian development. Front. Mol. Neurosci..

[39] Boukhatem I., Fleury S., Jourdi G., Lordkipanidzé M. (2024). The intriguing role of platelets as custodians of brain-derived neurotrophic factor. Research and Practice in Thrombosis and Haemostasis.

[40] Ornell F., Scherer J.N., Schuch J.B., Sordi A.O., Halpern S.C., Rebelatto F.P., Bristot G., Kapczinski F., Roglio V.S., Pechansky F., Kessler F.H.P., von Diemen L. (2023). Serum BDNF levels increase during early drug withdrawal in alcohol and crack cocaine addiction. Alcohol (Fayetteville, N.Y.).

[41] Zhou L., Xiong J., Ruan C.-S., Ruan Y., Liu D., Bao J.-J., Zhou X.-F. (2018). ProBDNF/p75NTR/sortilin pathway is activated in peripheral blood of patients with alcohol dependence. Transl. Psychiatry.

[42] Crews F.T., Nixon K. (2009). Mechanisms of neurodegeneration and regeneration in alcoholism. Alcohol Alcohol.

[43] Crews F.T., Nixon K. (2003). Alcohol, neural stem cells, and adult neurogenesis. Alcohol Res. Health: The Journal of the National Institute on Alcohol Abuse and Alcoholism.

[44] Fayard B., Loeffler S., Weis J., Vögelin E., Krüttgen A. (2005). The secreted brain-derived neurotrophic factor precursor pro-BDNF binds to TrkB and p75NTR but not to TrkA or TrkC. J. Neurosci. Res..

[45] Mowla S.J., Farhadi H.F., Pareek S., Atwal J.K., Morris S.J., Seidah N.G., Murphy R.A. (2001). Biosynthesis and post-translational processing of the precursor to brain-derived neurotrophic factor. J. Biol. Chem..

[46] Sakuragi S., Tominaga-Yoshino K., Ogura A. (2013). Involvement of TrkB- and p75(NTR)-signaling pathways in two contrasting forms of long-lasting synaptic plasticity. Sci. Rep..

[47] Han C., Ahn D., Hahm W., Nam J., Park Y., Lim S., Kim D.-J. (2016). Serum levels of growth factors in alcohol-dependent patients according to comorbid depressive symptoms. Clinical Psychopharmacology and Neuroscience: The Official Scientific Journal of the Korean College of Neuropsychopharmacology.

[48] Cavus S.Y., Dilbaz N., Darcin A.E., Eren F., Kaya H., Kaya O. (2012). Alterations in serum BDNF levels in early alcohol withdrawal and comparison with healthy controls. Klinik Psikofarmakoloji Bülteni-Bulletin of Clinical Psychopharmacology.

[49] Jiang H., Chen S., Li C., Lu N., Yue Y., Yin Y., Zhang Y., Zhi X., Zhang D., Yuan Y. (2017). The serum protein levels of the tPA-BDNF pathway are implicated in depression and antidepressant treatment. Transl. Psychiatry.

[50] Zhou L., Xiong J., Lim Y., Ruan Y., Huang C., Zhu Y., Zhong J., Xiao Z., Zhou X.-F. (2013). Upregulation of blood proBDNF and its receptors in major depression. J. Affect. Disord..

[51] Clergue-Duval V., Coulbault L., Questel F., Cabé N., Laniepce A., Delage C., Boudehent C., Bloch V., Segobin S., Naassila M., Pitel A.-L., Vorspan F. (2022). Alcohol withdrawal is an oxidative stress challenge for the brain : does it pave the way toward severe alcohol-related cognitive impairment?. Antioxidants.

[52] Schmidt P., Helten C., Soyka M. (2011). Predictive value of obsessive-compulsive drinking scale (OCDS) for outcome in alcohol-dependent inpatients : results of a 24-month follow-up study. Subst. Abuse Treat. Prev. Pol..

[53] Szuhany K.L., Otto M.W. (2020). Assessing BDNF as a mediator of the effects of exercise on depression. J. Psychiatr. Res..

