# Supplementary material for: Serum BDNF and pro-BDNF levels in alcohol use disorders according to depression status: An exploratory study of their evolution two months after withdrawal
Source: Heliyon. 2024 Oct 4;10(19):e38940. doi: 10.1016/j.heliyon.2024.e38940 (PMC11490827; doi:10.1016/j.heliyon.2024.e38940)
Supplement: Multimedia component 1 [file mmc1.pdf]

Données générales

NOM..**Prénom**..

NOM Patronymique..

Sexe ☐ **F** ☐ **M** Date de Naissance (jj/mm/aa) |__|__| |__|__| |__|__|

Adresse de résidence ....

....

Tél. domicile :..portable :..

Médecin généraliste traitant (nom-adr) : ....

....

Psychiatre traitant (nom-adr) : ....

....

Centre investigateur :

☐ **CHE** Limoges unité : ....

☐ **CHU** Limoges Service Hépato-gastro-entérologie

☐ **La Jonchère**

☐ CH **Vauclaire**

Nom du Praticien investigateur : ....

si Frais de transport :

	JO	J14	J28	M2	M4	M6
Dates						
montant						

Evolution des taux sériques de Brain Derived Neurotrophic Factor au cours des 6 mois suivant un sevrage chez des sujets alcoolo-dépendants en fonction de l'abstinence (BDNF et alcool)

Etude n° 2011-A00452-39

Cahier d'Observations

NUMERO DOSSIER

**centre**	**nom**	**prénom**	**date d'hospitalisation**	**année d'inclusion**
**E** = CH Esquirol Limoges<br>**U** = CHU Limoges<br>**J** = La Jonchère<br>**V** = C.H. Vauclaire	première lettre	première lettre	jjmm	A = 2011<br>B = 2012<br>C = 2013<br>D = 2014
I__I	I__I	I__I	I__I__I__I__I	I__I

Date de l'information ....I__I__I I__I__I I_2_I_0_I__I__I

☐ **Non inclus / inclus à tort** ☐ **Inclus**

Signature du consentement le................I__I__I I__I__I I_2_I_0_I__I__I

Nom de l'Investigateur Coordonnateur	*Nom et coordonnées de l'Investigateur :*
Docteur Philippe NUBUKPO<br>P.A.L. – CH Esquirol, 15 rue du Dr Marcland<br>87025 LIMOGES cedex	

**Récapitulatif** - *Dates*	***Prévisionnel***	***Réel***
**INCLUSION J0**		
**J 14** (±2 jours)		
**J 28** (±3 jours)		
**M 2** (±5 jours)		
**M 4** (±7 jours)		
**M 6** (±7 jours)		

Date Sortie d'étude

J0

- ☐ Vérification des critères d'inclusion et de non inclusion
- ☐ Diagnostic DSM-IV-TR selon les 5 axes
- ☐ Données biologiques
- ☐ Données socio-démographiques
- ☐ Données para-cliniques
- ☐ MINI
- ☐ Traitement prescrit au moment du sevrage
- ☐ HAM-A Hamilton Anxiety Scale
- ☐ MADRS
- ☐ Inventaire de dépression de Beck
- ☐ Auto-questionnaire AUDIT
- ☐ OCDS
- ☐ Tableau consommation

Département Recherche & Développement
Centre Hospitalier Esquirol, 15 rue du Docteur Marcland
87025 LIMOGES cedex
Tél. 05.55.43.11.00 fax 05.55.43.11.11 email : drd@ch-esquirol-limoges.fr

Vérification des critères d'inclusion et de non inclusion
Réponse nécessaire à tous les critères

Critères d'INCLUSION

Diagnostic de dépendance alcoolique (critères DSM-IV-TR) ☐ **oui** ☐ **non**

Demande de sevrage ☐ **oui** ☐ **non**

Si un « non » coché, ne pas compléter la suite

Critères de NON inclusion : aucune case « oui » ne doit être cochée

Age < 18 ans	☐ oui	☐ non
Co-morbidité neurologique caractérisée (Korsakoff) diagnostiquée	☐ oui	☐ non
Pathologie somatique évolutive grave (pancréatite, gastrite, hépatite chronique) et/ou à évolution fatale proche attendue dans l'année (cancer, cirrhose…)	☐ oui	☐ non
Incapacité à répondre aux questionnaires (non francophone, détérioration cognitive et intellectuelle, …)	☐ oui	☐ non
Grossesse	☐ oui	☐ non
Prise de traitement anti-rétroviral	☐ oui	☐ non
Protection juridique (tutelle, curatelle, sauvegarde de justice)	☐ oui	☐ non
Hospitalisation sous contrainte (hospitalisation d'office ou à la demande d'un tiers)	☐ oui	☐ non
Absence d'assurance sociale	☐ oui	☐ non
Résidence à plus de 80 km d'un centre investigateur	☐ oui	☐ non
Participation à une autre recherche biomédicale durant les 6 mois de l'étude	☐ oui	☐ non
Refus de participer<br>si oui, raison du refus :	☐ oui	☐ non

Si une seule case « oui » cochée, ne pas poursuivre le remplissage du dossier

Date...*Praticien référent*...*Unité Fonctionnelle*..

complétez la page suivante

Diagnostic DSM-IV-TR selon les 5 axes

Axe I - Troubles cliniques et autres situations qui peuvent faire l'objet d'un examen clinique (p38 mini dsm)

1- Code diagnostique F |__|__| . |__|__|

2- Comorbidité I *(éventuellement)* F |__|__| . |__|__|

3- Comorbidité II *(éventuellement)* F |__|__| . |__|__|

Axe II - Troubles de la personnalité - Retard mental *(éventuellement)* (p38 mini dsm)

1- Troubles de la personnalité F |__|__| . |__|__|

2- Retard mental F |__|__| . |__|__|

Axe III - Affections médicales générales - CIM 10 (*(éventuellement)*) (p39 mini dsm)

1- *En clair* .... |__| |__|__| . |__|__|

2- *En clair* .... |__| |__|__| . |__|__|

Axe IV - Problèmes psychosociaux et environnementaux – *cocher les seuls problèmes présents* (p41 mini dsm)

Problèmes avec le groupe de support social *spécifier* .... ☐ 1
Problèmes liés à l'environnement social *spécifier* .... ☐ 2
Problèmes d'éducation *spécifier* .... ☐ 3
Problèmes professionnels *spécifier* .... ☐ 4
Problèmes de logement *spécifier* .... ☐ 5
Problèmes économiques *spécifier* .... ☐ 6
Problèmes d'accès aux Services de santé *spécifier* .... ☐ 7
Problèmes avec les institutions judiciaires/pénales *spécifier* .... ☐ 8
Autres problèmes psychosociaux et environnementaux *spécifier* .... ☐ 9

Axe V - Echelles d'évaluation (p43 mini dsm) ***score de 0 à 100***

Echelle globale du fonctionnement ....|__|__|__|

Date ....

Praticien référent ....

Unité Fonctionnelle..

Données biologiques

PRELEVEMENTS *(cocher la case correspondant)*

Date	....			
Analyse	Support	Prélèvement au centre	Prélèvement dans l'unité	
			bilan d'Entrée	spécifique
GGT	*Tube héparinate bouchon vert*			
CDT	*Tube sec bouchon jaune*			
Aliquots	*Tube sec bouchon jaune*			
EtG urinaire	*Flacon urine*			
EtG capillaire	*Kit prélèvement cheveux noter si impossibilité*			

Recueil de l'Opposition à la conservation des résidus de prélèvements de sérum dans la cadre de la « collection biologique en psychiatrie » du CH Esquirol

Opposition exprimée : ☐ non.............. ☐ oui

Responsable de la collecte de l'information : ....

RESULTATS

GGT I__I__I__I__I U.I./L

CDT I__I__I , I__I__I %

EtG urinaire I__I__I__I__I , I__I__I mg/L

BDNF sérique I__I__I__I , I__I pg/mL

l'EtG capillaire I__I__I__I pg/mg

DETERMINATION DE L'IMC

Poids (kg) I__I__I__I Taille (cm) I__I__I__I

IMC (kg/cm²) I__I__I__I

Données socio-démographiques

Sexe ☐ F ☐ M Date de Naissance (mm/aa) |__|__| |__|__|

Mode de vie

☐ Chez les parents ou famille
☐ Seul
☐ Seul avec enfant(s)
☐ En couple sans enfant
☐ En couple avec enfant(s)
☐ En famille d'accueil
☐ En institution
☐ Sans information

Situation matrimoniale

☐ Célibataire
☐ Marié(e)
☐ PACS
☐ Divorcé(e)
☐ Veuf(ve)
☐ Sans information

Niveau d'études

☐ CAP-BEP - Technique
☐ baccalauréat
☐ université
☐ sans diplôme
☐ sans information

Activité professionnelle

☐ milieu ordinaire
☐ milieu protégé
☐ demandeur d'emploi
☐ sans activité
☐ retraité
☐ sans information

Type de ressources

☐ Ressources propres
☐ Ressources sociales
  ☐ AES
  ☐ AAH
  ☐ RSA
  ☐ Invalidité
  ☐ Autres
☐ Ressources familiales
☐ Sans ressources

Données para-cliniques

Année approximative du 1er contact avec un psychiatre ou une équipe psychiatrique : I__I__I__I__I

Nombre de sevrages précédents :

- au domicile I__I__I
- en institution I__I__I

Nombre de cures antérieures I__I__I

PRATIQUE D'ACTIVITÉ PHYSIQUE ☐ non ☐ oui
si oui :

type d'activité (inclure la marche si elle est un moyen de transport) ....

pratique intensive (club, compétition) ☐ oui ☐ non

temps moyen dans la semaine (heures) ....

MOTIVATION À L'ARRET

« Sur une échelle de 1 à 10, à quel point est-il important de modifier votre consommation d'alcool ? »

Réponse : I__I__I

TRAITEMENT
Instauration de traitement depuis moins de 3 mois : ☐ aucun ☐ antidépresseur ☐ neuroleptique

ALCOOL Consommation durant les 2 mois précédant le sevrage :
Compléter le calendrier (TLFB) sur les jours correspondant aux deux derniers mois.

nombre de verres totaux I__I__I__I__I

nombre de verres moyen par jour I__I__I

nombre de jours de consommation d'alcool I__I__I

nombre de verres consommés par jour de consommation I__I__I

consommation au moins 5 jours consécutifs ☐ non ☐ oui

consommation de 4/5 verres ou plus en une fois ☐ non ☐ oui

si la réponse à la question, est "oui", continuer avec la question suivante sauf si spécification autre. Si un diagnostic figure sur la ligne, l'entourer

			NON	OUI	diagnostic
**A**	A1				
	A2				
	A1 **ou** A2 = oui				si non, aller en B
	A3	a-b-c-d-e-f-g-			
	A4	y-a-il au moins **3 OUI** en **A3** ?<br>(ou 4 si A1 OU A2 EST COTEE NON)			EDM ACTUEL
	A5	a			
		b			
		**A5b** est-elle cotée **OUI** (1 ou + épisodes)?			EDM PASSE
**B**	B1				si non, aller en C
	B2				si oui, aller en C
	B3	a-b-c-d-e-f			
		y-a-t-il au moins **2 OUI** en **B3** ?			si non, aller en C
	B4	**B4** est-elle cotée **OUI** ?			DYSTHYMIE ACTUEL
**C**	C1				
	C2				
	C3				
	C4				
	C5				
	C6				
		si au moins **1 OUI**			RISQUE SUICIDAIRE ACTUEL
		C1 ou C2 ou C6 = OUI : LEGER			
		C3 ou (C2 + C6) = OUI : MOYEN			
		C4 ou C5 ou (C3 + C6) = OUI : ELEVE			

		**ACTUEL**	NON	OUI	NON	OUI	**PASSE**
**D**	D1a						D1p
	D2a						D2p
		D1 **ou** D2 sont-elles cotées **OUI** ?					D1 **ou** D2 sont-elles cotées **OUI** ?
	D3a	a-b-c-d-e-f-g					
		y-a-t-il au moins **3 OUI** en **D3**					
	D4	**D4** est cotée **NON** = EPISODE HYPOMAN. ACTUEL					**D4** est cotée **NON** = EPISODE HYPOMAN. PASSE, aller en D5
		**D4** est cotée **OUI** = EPISODE MANIAQUE ACTUEL					**D4** est cotée **OUI** = EPISODE MANIAQUE PASSE, aller en D7
	D5						
	D7						

			NON	OUI	diagnostic
**E**	E1				si **E1 = NON** ALLER à F1
	E2				si **E2 = NON**, ALLER à F1
	E3				si **E3 = NON** ALLER à F1
	E4	a-b-c-d-e-f-g-h-i-j-k-l-m			
	E5	y-a-t-il au moins **4 OUI** en **E4**			*trouble panique vie entiere*
		si **E5 = NON,** passer à **E7**			
	E6	si **E6= OUI**, passer à **F1**			*trouble panique actuel*
	E7	y-a-t-il 1, 2 ou 3 **OUI** en **E4**			*attaques paucisymptom. vie ent.*
**F**	F1	si **F1 = NON**, entourer NON en F2			ALLER EN G1
	F2	si F2=OUI			*agoraphobie « actuel »*
	**F2** est-elle cotée **NON** ? **et E6** est-elle cotée **OUI** ?				TROUBLE PANIQUE Sans Agoraphobie « ACTUEL »
	**F2** est-elle cotée **OUI** ? **et E6** est-elle cotée **OUI** ?				TROUBLE PANIQUE Avec Agoraphobie « ACTUEL »
	**F2** est-elle cotée **OUI** ? **et E5** est-elle cotée **NON** ?				AGORAPHOBIE sans atcds de Trouble Panique « ACTUEL »
**G**	G1				si non, aller en H
	G2				si non, aller en H
	G3				si non, aller en H
	G4	**G4** est-elle cotée **OUI** ?			PHOBIE SOCIALE ACTUEL
**H**	H1	si **H1 = NON,** passer à **H4**			
	H2	si **H2 = NON**, passer à **H4**			
	H3				
	H4				
		**H3** **ou** **H4** sont-elles cotées **OUI** ?			si **NON**, passer à **J1**
	H5				si **H2 = NON**, passer à **J1**
	H6	**H6** est-elle cotée **OUI** ?			TROUBLE OBS COMPULSIF
**J**	J1				si non, aller en J3
	J2	a-b-c-d-e-f-g			
		Y a-t-il au moins **3 OUI** en **J2** ?			DEPENDANCE ALCOOLIQUE ACTUEL, aller à K
	J3	a-b-c-d			
		**Y a-t-il au moins 1 OUI en J3 ?**			ABUS D'ALCOOL ACTUEL

T	T1	**TABAC**			Si Non aller en U
	**quantité**				
	**depuis combien d'années**				
	T2-3-4-5-6-				
	Y a-t-il au moins **2 OUI** en T2-3-4-5-6				DEPENDANCE nicotine ABUS
U	U1	**CAFE**			
	U2	**quantité / jour**			
	U3				
	U4	**SODA**			
		combien			

			NON	OUI	diagnostic
**K**	K1				Si NON aller en L
	K2	Si OUI, quelles substances :			
		Substance la + consommée :			
		Nbre jours/semaine			
		Quantité / semaine			
		a-b-c-d-e-f-g-h			
		Y A-T-IL AU MOINS **3 OUI** EN **K2** ?			DEPENDANCE A UNE SUBSTANCE « ACTUEL »
	K3	le patient présente-t-il une dép. pour la subst?			si oui, aller en L
		a-b-c-d			
		Y a-t-il au moins **1 OUI** en **K3 ?**			ABUS DE SUBSTANCE ACTUEL

			a			b			
			NON	OUI	BIZARRE	NON	OUI	BIZARRE	
**L**	L1	a :							
		b							si oui bizarre, aller en L6a
	L2	a :							
		b							si oui bizarre, aller en L6a
	L3	a :							
		b							si oui bizarre, aller en L6a
	L4	a :							
		b							si oui bizarre, aller en L6a
	L5	a :							
		b							
	L6	a :							
		b							si oui, aller en L8b
	L7	a :							
		b							
	L8	b							
	L9	b							
	L10	b							

			NON	OUI	diagnostic
	L11	DE **L1 A L10**, y a-t-il a moins<br>1 QUESTION " b " COTEE BIZARRE ou<br>2 QUESTIONS " b " COTEES OUI (NON BIZ) ?			SYNDROME PSYCHOTIQUE ACTUEL
	L12	de **L1 à L7**, y-a-t-il au moins<br>1 question " a " cotée **BIZARRE ou**<br>2 questions " a " cotées OUI (NON BIZ.) ?<br>(les 2 sympt. survenus en même temps) **ou**<br>**L11** est-elle cotée **OUI** ?			SYNDROME PSYCHOTIQUE VIE ENTIERE
	L13-a	si **L11** est cotée **OUI** ou si il y a au moins **1 OUI** de L1 à L7<br>le patient présente-t-il<br>UN EPISODE DEPRESSIF MAJEUR ? ou<br>UN EPISODE MANIAQUE ?			si non, aller en M
	L13-b	**L13b** EST-ELLE COTEE **OUI ?**			TROUBLE DE L'HUMEUR +avec caract. psychotiques actuel

			NON	OUI	diagnostic
**M**	M1	a – **Taille**			
		b – **Poids le + faible / 3 mois**			
		c			si non, aller en N
	M2				si non, aller en N
	M3				si non, aller en N
	M4	a-b-c			
	M5	y-a-t-il au moins **1 OUI** en **M4**			si non, aller en N
	M6				si non, aller en N
		POUR LES FEMMES: M5 et M6 sont-elles cotées oui ?			ANOREXIE MENTALE ACTUEL
		POUR LES HOMMES : M5 est-elle cotée oui ?			
**N**	N1				si non, aller en O
	N2				si non, aller en O
	N3				si non, aller en O
	N4				si non, aller en O
	N5				si non, aller en O
	N6				si non, passer à N8
	N7				
	N8	**N5** est-elle cotée **OUI et N7** cotée **NON** (ou non cotée) ?			BOULIMIE ACTUEL
		**N7** est-elle cotée **OUI** ?			ANOREXIE MENTALE binge eating/purging type
**O**	O1	a			si non, fin
		b			si non, fin
	O2				si non, fin
	O3	a-b-c-d-e-f			
		y a-t-il au moins **3 oui en O3 ?**			ANXIETE GENERALISEE ACTUEL

n° observation sphinx |__| |__| |__| |__|

Date de l'entretien ....

Fait par : ....

Traitement prescrit au moment du sevrage

ou feuille d'impression Informatique

NOM DU TRAITEMENT	Dosage	conditionnement	Unité	si besoin	matin	midi	soir	coucher	Classe de molécules
									AD = antidépresseur<br>NL = neuroleptique<br>EPI = anti-épileptique<br>ANT = antalgique<br>INF = anti-inflammatoire<br>D = divers<br>AX = anxiolytiques<br>A = aucun
**EXEMPLE**									
**Effexor**	**37,5**	**LP**	**cp**						AD
**Risperdal**	**2**								NL
**Tercian**			**Gttes**						NL

HAM-A Hamilton Anxiety Scale

Date :

Cotation : 0=rien 1=faible 2=modérée 3=forte 4=très forte

		0	1	2	3	4
1	**Etat d'inquiétude :** Tracas, anticipation du pire, anticipation effrayante, irritabilité	☐	☐	☐	☐	☐
2	**Tension :** Impression de tension, fatigabilité, réactions de sursaut, facilité à fondre en larmes, tremblements, impression de ne pas pouvoir tenir en place, impossibilité de se détendre.	☐	☐	☐	☐	☐
3	**Etat dépressif :** Perte d'intérêt, manque de plaisir dans les loisirs, dépression, réveil matinal, agitation diurne	☐	☐	☐	☐	☐
4	**Conduite pendant l'entrevue :** Oubli, tremblement des mains, front plissé, visage tendu, soupirs ou respiration courte, pâleur du visage, déglutition, rots, sursauts tendineux, mydriase, exophtalmie.	☐	☐	☐	☐	☐
5	**Peurs – Frayeurs :** De l'obscurité, des étrangers, d'être seul, des animaux, de la circulation (auto), des foules.	☐	☐	☐	☐	☐
6	**Intellect :** Difficulté de concentration, mémoire défaillante.	☐	☐	☐	☐	☐
7	**Insomnie :** Difficulté de s'endormir, sommeil intermittent, sommeil non réparateur avec fatigue au réveil, rêves, cauchemars, terreurs nocturnes	☐	☐	☐	☐	☐
8	**Symptômes autonomes :** Bouche sèche, érithrose, pâleur, tendance à transpirer, étourdissements, maux de tête pendant les périodes de tension, cheveux qui se dressent.	☐	☐	☐	☐	☐
9	**Symptômes génito-urinaires :** Fréquence de la miction, urgence de la miction, aménorrhée, ménorragie, frigidité, éjaculation précoce, baisse ou perte de la lilbido.	☐	☐	☐	☐	☐
10	**Symptômes cardio-vasculaires :** Tachycardie, palpitations, douleur thoracique, sensation des pulsations artérielles, impression de faiblesse (ou d'évanouissement), arythmie (extra-systolie).	☐	☐	☐	☐	☐
11	**Somatique – sensorielle :** Troubles de la vision (flou), bouffées de chaleur et sueurs froides, impressions de faiblesse (physique), sensations de picotement.	☐	☐	☐	☐	☐
12	**Somatique – musculaire :** Douleurs musculaires, sursauts musculaires, raideurs musculaires, secousses myocloniques, grincements des dents, voix hésitante (tremblotante), augmentation du tonus musculaire.	☐	☐	☐	☐	☐
13	**Symptômes gastro-intestinaux :** Difficulté de déglutition, gaz, douleurs abdominales, sensations de brûlure, ballonnement abdominal, nausée, vomissements, borborygmes, relâchement des sphincters, perte de poids, constipation.	☐	☐	☐	☐	☐
14	**Symptômes respiratoires :** Sensation d'oppression, impression d'étouffement, soupirs, dyspnée.	☐	☐	☐	☐	☐

MADRS

Montgomery Asberg Depression Rating Scale

Date :

	0	1	2	3	4	5	6
**Tristesse apparente**	☐ Pas de tristesse	☐	☐ Semble découragé	☐	☐ Paraît triste et malheureux la plupart du temps	☐	☐ Semble malheureux tout le temps
**Tristesse exprimée**	☐ Tristesse occasionnelle (circonstances)	☐	☐ Triste, se déride sans difficulté	☐	☐ Sentiment envahissant de tristesse	☐	☐ Tristesse, désespoir permanent
**Tension intérieure**	☐ Calme, tension intérieure passagère	☐	☐ Irritabilité, malaise mal défini occasionnels	☐	☐ Sentiments continuels de tension, panique intermittente	☐	☐ Effroi, angoisse, panique envahissants
**Réduction de sommeil**	☐ Dort comme d'habitude	☐	☐ Légère difficulté à s'endormir	☐	☐ Sommeil réduit d'environ 2 heures	☐	☐ Moins de 2 ou 3 heures de sommeil
**Réduction de l'appétit**	☐ Appétit normal ou augmenté	☐	☐ Appétit légèrement réduit	☐	☐ Pas d'appétit	☐	☐ Ne mange que si on le persuade
**Lassitude**	☐ Pas de difficultés	☐	☐ Difficultés à commencer des activités	☐	☐ Les activités routinières sont poursuivies avec effort	☐	☐ Grande lassitude. Nécessité d'aide
**Incapacité à ressentir**	☐ Intérêt normal pour l'entourage	☐	☐ Capacité réduite à prendre plaisir	☐	☐ Perte d'intérêt, perte de sentiments	☐	☐ Sentiment de paralysie émotionnelle
**Difficultés de concentration**	☐ Pas de difficultés	☐	☐ Difficultés occasionnelles	☐	☐ Difficultés à maintenir son attention	☐	☐ Incapacité à se concentrer
**Pensées pessimistes**	☐ Pas de pensées pessimistes	☐	☐ Idées intermittentes d'échec	☐	☐ Auto-accusation, culpabilité persistante	☐	☐ Idées délirantes de ruine, d'auto-accusations
**Idées de suicide**	☐ Jouit de la vie	☐	☐ Fatigué de la vie, idées de suicide passagères	☐	☐ Il vaudrait mieux être mort, idées de suicide courantes	☐	☐ Projets explicites de suicide

Inventaire de dépression de Beck

Traduction française : P. PICHOT

Date :

Doit être rempli par le participant lui-même – à compléter sur place

Instructions : *Ce questionnaire comporte 13 séries (A à M) de quatre propositions. Pour chaque série, cochez le numéro à droite qui décrit le mieux votre état actuel*

A	Je ne me sens pas triste	☐	0	**A**
	Je me sens cafardeux ou triste	☐	1	
	Je me sens tout le temps cafardeux ou triste, et je n'arrive pas à en sortir	☐	2	
	Je suis si triste et si malheureux que je ne peux pas le supporter	☐	3	
B	Je ne suis pas particulièrement découragé ni pessimiste au sujet de l'avenir	☐	0	**B**
	J'ai un sentiment de découragement au sujet de l'avenir	☐	1	
	Pour mon avenir, je n'ai aucun motif d'espérer	☐	2	
	Je ne sens qu'il y a aucun espoir pour mon avenir, et que la situation ne peut s'améliorer	☐	3	
C	Je n'ai aucun sentiment d'échec dans ma vie	☐	0	**C**
	J'ai l'impression que j'ai échoué dans ma vie plus que la plupart des gens	☐	1	
	Quand je regarde ma vie passée, tout ce que j'y découvre n'est qu'échecs	☐	2	
	J'ai un sentiment d'échecs complet dans toute ma vie personnelle (dans mes relations avec mes parents, mon mari, ma femme, mes enfants)	☐	3	
D	Je ne me sens pas particulièrement insatisfait	☐	0	**D**
	Je ne sais pas profiter agréablement des circonstances	☐	1	
	Je ne tire plus aucune satisfaction de quoi que ce soit	☐	2	
	Je suis mécontent de tout	☐	3	
E	Je ne me sens pas coupable	☐	0	**E**
	Je me sens mauvais ou indigne une bonne partie du temps	☐	1	
	Je me sens coupable	☐	2	
	Je me juge très mauvais, et j'ai l'impression que je ne vaux rien	☐	3	
F	Je ne suis pas déçu par moi-même	☐	0	**F**
	Je suis déçu par moi-même	☐	1	
	Je me dégoûte moi-même	☐	2	
	Je me hais	☐	3	
G	Je ne pense pas à me faire du mal	☐	0	**G**
	Je pense que la mort me libérerait	☐	1	
	J'ai des plans précis pour me suicider	☐	2	
	Si je le pouvais, je me tuerais	☐	3	
H	Je n'ai pas perdu l'intérêt pour les autres gens	☐	0	**H**
	Maintenant, je m'intéresse moins aux autres gens qu'autrefois	☐	1	
	J'ai perdu tout l'intérêt que je portais aux autres gens, et j'ai peu de sentiments pour eux	☐	2	
	J'ai perdu tout intérêt pour les autres, et ils m'indiffèrent totalement	☐	3	
I	Je suis capable de me décider aussi facilement que de coutume	☐	0	**I**
	J'essaie de ne pas avoir à prendre de décision	☐	1	
	J'ai de grandes difficultés à prendre des décisions	☐	2	
	Je ne suis plus capable de prendre la moindre décision	☐	3	
J	Je n'ai pas le sentiment d'être plus laid qu'avant	☐	0	**J**
	J'ai peur de paraître vieux ou disgracieux	☐	1	
	J'ai l'impression qu'il y a un changement permanent dans mon apparence physique, qui me fait paraître disgracieux	☐	2	
	J'ai l'impression d'être laid et repoussant	☐	3	
K	Je travaille aussi facilement qu'auparavant	☐	0	**K**
	Il me faut aussi un effort supplémentaire pour commencer à faire quelque chose	☐	1	
	Il faut que je fasse un très grand effort pour faire quoi que ce soit	☐	2	
	Je suis incapable de faire le moindre effort	☐	3	
L	Je ne suis pas plus fatigué que d'habitude	☐	0	**L**
	Je suis fatigué plus facilement que d'habitude	☐	1	
	Faire quoi que ce soit me fatigue	☐	2	
	Je suis incapable de faire le moindre travail	☐	3	
M	Mon appétit est toujours aussi bon	☐	0	**M**
	Mon appétit n'est pas aussi bon que d'habitude	☐	1	
	Mon appétit est beaucoup moins bon maintenant	☐	2	
	Je n'ai plus du tout d'appétit	☐	3	

Merci

**Auto-questionnaire AUDIT**	Date :

A remplir par le participant lui-même – à compléter sur place

Instructions : *Cochez la case adaptée pour chacune des 10 questions*

	score	0	1	2	3	4
1	1. Quelle est la fréquence de votre consommation d'alcool ?	☐Jamais	☐ 1 fois par mois ou moins	☐ 2 à 4 fois/mois	☐ 2 à 3 fois par semaine	☐ Au moins 4 fois par semaine
2	Combien de verres contenant de l'alcool consommez-vous un jour typique où vous buvez ?	☐1 ou 2	☐3 ou 4	☐5 ou 6	☐7 ou 8	☐10 ou plus
3	Avec quelle fréquence buvez-vous six verres ou davantage lors d'une occasion particulière ?	☐Jamais	☐Moins d'une fois par mois	☐Une fois par mois	☐Une fois par semaine	☐Tous les jours ou presque
4	Au cours de l'année écoulée, combien de fois avez-vous constaté que vous n'étiez plus capable de vous arrêter de boire une fois que vous aviez commencé ?	☐Jamais	☐Moins d'une fois par mois	☐Une fois par mois	☐Une fois par semaine	☐Tous les jours ou presque
5	Au cours de l'année écoulée, combien de fois votre consommation d'alcool vous a-t-elle empêché de faire ce qui était normalement attendu de vous ?	☐Jamais	☐Moins d'une fois par mois	☐Une fois par mois	☐Une fois par semaine	☐Tous les jours ou presque
6	Au cours de l'année écoulée, combien de fois avez-vous eu besoin d'un premier verre pour pouvoir démarrer après avoir beaucoup bu la veille ?	☐Jamais	☐Moins d'une fois par mois	☐Une fois par mois	☐Une fois par semaine	☐Tous les jours ou presque
7	Au cours de l'année écoulée, combien de fois avez-vous eu un sentiment de culpabilité ou des remords après avoir bu ?	☐Jamais	☐Moins d'une fois par mois	☐Une fois par mois	☐Une fois par semaine	☐Tous les jours ou presque
8	Au cours de l'année écoulée, combien de fois avez-vous été incapable de vous rappeler ce qui s'était passée la soirée précédente parce que vous aviez bu ?	☐Jamais	☐Moins d'une fois par mois	☐Une fois par mois	☐Une fois par semaine	☐Tous les jours ou presque
9	Avez-vous été blessé ou quelqu'un d'autre a-t-il été blessé parce que vous aviez bu ?	☐Non		☐Oui, mais pas au cours de l'année écoulée		☐Oui, au cours de l'année
10	Un parent, un ami, un médecin ou un autre soignant s'est-il inquiété de votre consommation d'alcool ou a-t-il suggéré que vous la réduisiez ?	☐Non		☐Oui, mais pas au cours de l'année écoulée		☐Oui, au cours de l'année

A remplir par le participant lui-même

OCDS	Date :

Instructions : Les questions suivantes concernent votre consommation d'alcool et votre désir de contrôler cette consommation dans les **7 derniers jours**.
Veuillez **entourer** le chiffre en face de la réponse qui s'applique le mieux à votre état.

Q1 Lorsque vous ne buvez pas d'alcool, combien de votre temps est occupé par des idées, pensées, impulsions ou images liées à la consommation d'alcool ?

0 ☐ A aucun moment
1 ☐ moins d'une heure par jour
2 ☐ de 1 à 3 heures par jour
3 ☐ de 4 à 8 heures par jour
4 ☐ plus de 8 heures par jour

Q2 A quelle fréquence ces pensées surviennent-elles ?

0 ☐ jamais
1 ☐ pas plus de 8 fois par jour
2 ☐ plus de 8 fois par jour, mais pendant la plus grande partie de la journée je n'y pense pas
3 ☐ plus de 8 fois par jour et pendant la plus grande partie de la journée
4 ☐ ces pensées sont trop nombreuses pour être comptées et il ne se passe que rarement une heure sans que plusieurs de ces idées ne surviennent

Q3 A quel point ces idées, pensées, impulsions ou images liées à la consommation d'alcool interfèrent-elles avec votre activité sociale ou professionnelle (ou votre fonction) **? Y a-t-il quelque chose que vous ne faites pas ou ne pouvez pas faire à cause d'elles ?** (si vous ne travaillez pas actuellement, à quel point vos capacités seraient-elles atteintes si vous travailliez ?)

0 ☐ Les pensées relatives à la consommation d'alcool n'interfèrent jamais. Je peux fonctionner normalement
1 ☐ Les pensées relatives à la consommation d'alcool interfèrent légèrement avec mes activités sociales ou professionnelles, mais mes performances globales n'en sont pas affectées
2 ☐ Les pensées relatives à la consommation d'alcool interfèrent réellement avec mes activités sociales ou professionnelles, mais je peux encore m'en arranger
3 ☐ Les pensées relatives à la consommation d'alcool affectent de façon importante mes activités sociales ou professionnelles
4 ☐ Les pensées relatives à la consommation d'alcool bloquent mes activités sociales ou professionnelles

Q4 Quelle est l'importance de la détresse ou de la perturbation que ces idées, pensées, impulsions ou images liées à la consommation d'alcool génèrent lorsque vous ne buvez pas?

0 ☐ Aucune
1 ☐ Légère, peu fréquente et pas trop dérangeante
2 ☐ Modérée, fréquente et dérangeante mais encore gérable
3 ☐ Sévère, très fréquente et très dérangeante
4 ☐ Extrême, presque constante et bloquant les capacités

Q5 Lorsque vous ne buvez pas, à quel point faites-vous des efforts pour résister à ces pensées ou essayer de les repousser ou de les détourner de votre attention quand elles entrent dans votre esprit? (Evaluez vos efforts faits pour résister à ces pensées, et non votre succès ou votre échec à les contrôler réellement)

0 ☐ Mes pensées sont si minimes que je n'ai pas besoin de faire d'effort pour y résister. Si j'ai de telles pensées, je fais toujours l'effort d'y résister
1 ☐ J'essaie d'y résister la plupart du temps
2 ☐ Je fais quelques efforts pour y résister
3 ☐ Je me laisse aller à toutes ces pensées sans essayer de les contrôler, mais je le fais avec quelque hésitation
4 ☐ Je me laisse aller complètement et volontairement à toutes ces pensées

Q6 Lorsque vous ne buvez pas, à quel point arrivez-vous à arrêter ces pensées ou à vous en détourner ?

0 ☐ Je réussis complètement à arrêter ou à me détourner de telles pensées
1 ☐ Je suis d'habitude capable d'arrêter ces pensées ou de me détourner d'elles avec quelques efforts et de la concentration
2 ☐ Je suis parfois capable d'arrêter de telles pensées ou de m'en détourner
3 ☐ Je n'arrive que rarement à arrêter de telles pensées et ne peux m'en détourner qu'avec difficulté
4 ☐ Je n'arrive que rarement à me détourner de telles pensées même momentanément

Q7 Combien de verres de boissons alcooliques buvez-vous par jour ?
0 ☐ Aucun
1 ☐ Moins d'un verre par jour
2 ☐ De 1 à 2 verres par jour
3 ☐ De 3 à 7 verres par jour
4 ☐ 8 verres ou plus par jour

Q8 Combien de jours par semaine buvez-vous de l'alcool ?
0 ☐ Aucun
1 ☐ Pas plus d'un jour par semaine
2 ☐ De 2 à 3 j ours par semaine
3 ☐ De 4 à 5 j ours par semaine
4 ☐ De 6 à 7 j ours par semaine

Q9 A quel point votre consommation d'alcool interfère-t-elle avec votre activité professionnelle ? Existe-t-il des choses que vous ne faites pas ou ne pouvez pas faire à cause de cette consommation ? (Si vous ne travaillez pas actuellement, à quel point vos capacités professionnelles seraient-elles affectées si vous travailliez ?)
0 ☐ Le fait de boire n'interfère jamais — je peux fonctionner normalement
1 ☐ Le fait de boire interfère légèrement avec mon activité professionnelle mais l'ensemble de mes capacités n'en est pas affecté
2 ☐ Le fait de boire interfère de manière certaine avec mon activité professionnelle, mais je peux m'en arranger
3 ☐ Le fait de boire affecte de façon importante mon activité professionnelle
4 ☐ Les problèmes d'alcool bloquent mes capacités de travail

Q10 A quel point votre consommation d'alcool interfère-t-elle avec votre activité sociale ? Existe-t-il des choses que vous ne faites pas ou ne pouvez pas faire à cause de cette consommation ?
0 ☐ Le fait de boire n'interfère jamais —je peux fonctionner normalement
1 ☐ Le fait de boire interfère légèrement avec mes activités sociales, mais l'ensemble de mes capacités n'est pas affecté
2 ☐ Le fait de boire interfère de manière certaine avec mes activités sociales, mais je peux encore m'en arranger
3 ☐ Le fait de boire affecte de façon importante mes activités sociales
4 ☐ Les problèmes d'alcool bloquent mes activités sociales

Q11 Si l'on vous empêchait de boire de l'alcool quand vous désirez prendre un verre, à quel point seriez-vous anxieux ou énervé ?
0 ☐ Je n'éprouverais ni anxiété ni irritation
1 ☐ Je ne deviendrais que légèrement anxieux ou irrité
2 ☐ L'anxiété ou l'irritation augmenterait mais resterait contrôlable
3 ☐ J'éprouverais une augmentation d'anxiété ou d'irritation très importante et dérangeante
4 ☐ J'éprouverais une anxiété ou une irritation très invalidante

Q12 A quel point faites-vous des efforts pour résister à la consommation de boissons alcooliques? (Evaluez uniquement vos efforts pour y résister et non votre succès ou votre échec à réellement contrôler cette consommation)
0 ☐ Ma consommation est si minime que je n'ai pas besoin d'y résister — si je bois, je fais l'effort de toujours y résister
1 ☐ J'essaie d'y résister la plupart du temps
2 ☐ Je fais quelques efforts pour y résister
3 ☐ Je me laisse aller presque à chaque fois sans essayer de contrôler ma consommation d'alcool, mais je le fais avec un peu d'hésitation
4 ☐ Je me laisse aller complètement et volontairement à la boisson

Q13 A quel point vous sentez-vous poussé à consommer des boissons alcooliques ?
0 ☐ Je ne me sens pas poussé du tout
1 ☐ Je me sens faiblement poussé à boire
2 ☐ Je me sens fortement poussé à boire
3 ☐ Je me sens très fortement poussé à boire
4 ☐ Le désir de boire est entièrement involontaire et me dépasse

Q14 Quel contrôle avez-vous sur votre consommation d'alcool ?
0 ☐ J'ai un contrôle total
1 ☐ Je suis habituellement capable d'exercer un contrôle volontaire sur elle
2 ☐ Je ne peux la contrôler qu'avec difficulté
3 ☐ Je dois boire et je ne peux attendre de boire qu'avec difficulté
4 ☐ Je suis rarement capable d'attendre de boire même momentanément

Evolution des taux sériques de Brain Derived Neurotrophic Factor au cours des 6 mois suivant un sevrage chez des sujets alcoolo-dépendants en fonction de l'abstinence (BDNF et alcool)

Etude n° 2011-A00452-39

Cahier d'Observations M2

N° DOSSIER I__I I__I__I I__I__I__I__I__I I__I

Date de la visite de suivi I__I__I I__I__I I_2_I_0_I__I__I
à reporter sur la 1ère page de J0

Lieu..

- ☐ Prélèvement sanguin
- ☐ Prélèvement urines
- ☐ Données para-cliniques
- ☐ MINI
- ☐ Traitement
- ☐ Inventaire de dépression de Beck
- ☐ OCDS
- ☐ HAM-A Hamilton Anxiety Scale
- ☐ MADRS
- ☐ Tableau consommation

PRELEVEMENTS *(cocher la case correspondant)*

Date	....			
Analyse	Support	Prélèvement au DRD	Prélèvement dans l'unité	
			bilan Hospitalier	spécifique
GGT	*Tube héparinate bouchon vert*			
CDT	*Tube sec bouchon jaune*			
Aliquots	*Tube sec bouchon jaune*			
EtG urinaire	*Flacon urine*			
EtG capillaire	*Kit prélèvement cheveux*			

RESULTATS

GGT I__I__I__I__I U.I./L

CDT I__I__I , I__I__I %

EtG urinaire I__I__I__I__I , I__I__I mg/L

BDNF sérique I__I__I__I , I__I pg/mL

DETERMINATION DE L'IMC

Poids (kg) I__I__I__I Taille (cm) I__I__I__I

IMC (kg/cm²) I__I__I__I

Données para-cliniques

PRATIQUE D'ACTIVITÉ PHYSIQUE ☐ non ☐ oui
si oui :
type d'activité (inclure la marche si elle est un moyen de transport) ....
pratique intensive (club, compétition) ☐ oui ☐ non
temps moyen dans la semaine (heures) ....

PATHOLOGIE SOMATIQUE ☐ non ☐ oui laquelle :

SOINS REÇUS depuis le dernier suivi

☐ Hospitalisation(s) CHE ☐ HC ☐ HJ motif ....
☐ en lien avec l'étude

☐ Autre(s) hospitalisation(s) motif ....
☐ en lien avec l'étude

☐ visite au médecin généraliste
☐ visite au médecin psychiatre
☐ groupe de parole ☐ autre

ALCOOL Consommation depuis le dernier suivi :
Compléter le calendrier (TLFB) sur les jours correspondants

nombre de verres totaux I__I__I__I__I

nombre de verres moyen par jour I__I__I

nombre de jours de consommation d'alcool I__I__I

nombre de verres consommés par jour de consommation I__I__I

ABSTINENCE ☐ non ☐ oui

Si non abstinent :

☐ partiel
(consommation d'alcool irrégulière / intermittente (< 5 jrs consécutifs)
OU = consommation occasionnelle <4/5 verres à chaque fois).

☐ rechute
(consommation d'alcool répétée au moins 5 jrs consécutifs
OU = consommation occasionnelle > 4/5 verres à chaque fois).

si la réponse à la question, est "oui", continuer avec la question suivante sauf si spécification autre. Si un diagnostic figure sur la ligne, l'entourer

			NON	OUI	diagnostic
**A**	A1				
	A2				
	A1 **ou** A2 = oui				si non, aller en B
	A3	a-b-c-d-e-f-g-			
	A4	y-a-il au moins **3 OUI** en **A3** ?<br>(ou 4 si A1 OU A2 EST COTEE NON)			EDM ACTUEL
	A5	a			
		b			
		**A5b** est-elle cotée **OUI** (1 ou + épisodes)?			EDM PASSE
**B**	B1				si non, aller en C
	B2				si oui, aller en C
	B3	a-b-c-d-e-f			
		y-a-t-il au moins **2 OUI** en **B3** ?			si non, aller en C
	B4	**B4** est-elle cotée **OUI** ?			DYSTHYMIE ACTUEL
**C**	C1				
	C2				
	C3				
	C4				
	C5				
	C6				
		si au moins **1 OUI**			RISQUE SUICIDAIRE ACTUEL
		C1 ou C2 ou C6 = OUI : LEGER			
		C3 ou (C2 + C6) = OUI : MOYEN			
		C4 ou C5 ou (C3 + C6) = OUI : ELEVE			

			NON	OUI	NON	OUI	
**D**		**ACTUEL**	NON	OUI	NON	OUI	**PASSE**
	D1a						D1p
	D2a						D2p
		D1 **ou** D2 sont-elles cotées **OUI** ?					D1 **ou** D2 sont-elles cotées **OUI** ?
	D3a	a-b-c-d-e-f-g					
		y-a-t-il au moins **3 OUI** en **D3**					
	D4	**D4** est cotée **NON** = EPISODE HYPOMAN. ACTUEL					**D4** est cotée **NON** = EPISODE HYPOMAN. PASSE, aller en D5
		**D4** est coté **OUI** = EPISODE MANIAQUE ACTUEL					**D4** est cotée **OUI** = EPISODE MANIAQUE PASSE, aller en D7
	D5						
	D7						

			NON	OUI	diagnostic
**E**	E1				si **E1** = **NON** ALLER à F1
	E2				si **E2** = **NON**, ALLER à F1
	E3				si **E3** = **NON** ALLER à F1
	E4	a-b-c-d-e-f-g-h-i-j-k-l-m			
	E5	y-a-t-il au moins **4 OUI** en **E4**			*trouble panique vie entiere*
		si **E5** = **NON**, passer à **E7**			
	E6	si **E6= OUI**, passer à **F1**			*trouble panique actuel*
	E7	y-a-t-il 1, 2 ou 3 **OUI** en **E4**			*attaques paucisymptom. vie ent.*
**F**	F1	si **F1** = **NON**, entourer NON en F2			ALLER EN G1
	F2	si F2=OUI			*agoraphobie « actuel »*
	**F2** est-elle cotée **NON** ? **et E6** est-elle cotée **OUI** ?				TROUBLE PANIQUE Sans Agoraphobie « ACTUEL »
	**F2** est-elle cotée **OUI** ? **et E6** est-elle cotée **OUI** ?				TROUBLE PANIQUE Avec Agoraphobie « ACTUEL »
	**F2** est-elle cotée **OUI** ? **et E5** est-elle cotée **NON** ?				AGORAPHOBIE sans atcds de Trouble Panique « ACTUEL »
**G**	G1				si non, aller en H
	G2				si non, aller en H
	G3				si non, aller en H
	G4	**G4** est-elle cotée **OUI** ?			PHOBIE SOCIALE ACTUEL
**H**	H1	si **H1** = **NON**, passer à **H4**			
	H2	si **H2** = **NON**, passer à **H4**			
	H3				
	H4				
		**H3** **ou** **H4** sont-elles cotées **OUI** ?			si **NON**, passer à **J1**
	H5				si **H2** = **NON**, passer à **J1**
	H6	**H6** est-elle cotée **OUI** ?			TROUBLE OBS COMPULSIF
**J**	J1				si non, aller en J3
	J2	a-b-c-d-e-f-g			
		Y a-t-il au moins **3 OUI** en **J2** ?			DEPENDANCE ALCOOLIQUE ACTUEL, aller à K
	J3	a-b-c-d			
		**Y a-t-il au moins 1 OUI en J3 ?**			ABUS D'ALCOOL ACTUEL

			NON	OUI	
T	T1	**TABAC**			Si Non aller en U
	**quantité**				
	**depuis combien d'années**				
	T2-3-4-5-6-				
	Y a-t-il au moins **2 OUI** en T2-3-4-5-6				DEPENDANCE nicotine ABUS
U	U1	**CAFE**			
	U2	**quantité / jour**			
	U3				
	U4	**SODA**			
		combien			

			NON	OUI	diagnostic
**K**	K1				Si NON aller en L
		Si OUI, quelles substances :			
		Substance la + consommée :			
	K2	Nbre jours/semaine			
		Quantité / semaine			
		a-b-c-d-e-f-g-h			
		Y A-T-IL AU MOINS **3 OUI** EN **K2** ?			DEPENDANCE A UNE SUBSTANCE « ACTUEL »
	K3	le patient présente-t-il une dép. pour la subst?			si oui, aller en L
		a-b-c-d			
		Y a-t-il au moins **1 OUI** en **K3 ?**			ABUS DE SUBSTANCE ACTUEL

			a NON	a OUI	a BIZARRE	b NON	b OUI	b BIZARRE	
**L**	L1	a :							
		b							si oui bizarre, aller en L6a
	L2	a :							
		b							si oui bizarre, aller en L6a
	L3	a :							
		b							si oui bizarre, aller en L6a
	L4	a :							
		b							si oui bizarre, aller en L6a
	L5	a :							
		b							
	L6	a :							
		b							si oui, aller en L8b
	L7	a :							
		b							
	L8	b							
	L9	b							
	L10	b							

			NON	OUI	diagnostic
	L11	DE **L1 A L10**, y a-t-il a moins<br>1 QUESTION " b " COTEE BIZARRE ou<br>2 QUESTIONS " b " COTEES OUI (NON BIZ) ?			SYNDROME PSYCHOTIQUE ACTUEL
	L12	de **L1 à L7**, y-a-t-il au moins<br>1 question " a " cotée **BIZARRE ou**<br>2 questions " a " cotées OUI (NON BIZ.) ?<br>(les 2 sympt. survenus en même temps) **ou**<br>**L11** est-elle cotée **OUI** ?			SYNDROME PSYCHOTIQUE VIE ENTIERE
	L13-a	si **L11** est cotée **OUI** ou si il y a au moins **1 OUI** de L1 à L7<br>le patient présente-t-il<br>UN EPISODE DEPRESSIF MAJEUR ? ou<br>UN EPISODE MANIAQUE ?			si non, aller en M
	L13-b	**L13b** EST-ELLE COTEE **OUI ?**			TROUBLE DE L'HUMEUR +avec caract. psychotiques actuel

			NON	OUI	diagnostic
**M**	M1	a – **Taille**			
		b – **Poids le + faible / 3 mois**			
		c			si non, aller en N
	M2				si non, aller en N
	M3				si non, aller en N
	M4	a-b-c			
	M5	y-a-t-il au moins **1 OUI** en **M4**			si non, aller en N
	M6				si non, aller en N
		POUR LES FEMMES: M5 et M6 sont-elles cotées oui ?			ANOREXIE MENTALE ACTUEL
		POUR LES HOMMES : M5 est-elle cotée oui ?			
**N**	N1				si non, aller en O
	N2				si non, aller en O
	N3				si non, aller en O
	N4				si non, aller en O
	N5				si non, aller en O
	N6				si non, passer à N8
	N7				
	N8	**N5** est-elle cotée **OUI et N7** cotée **NON** (ou non cotée) ?			BOULIMIE ACTUEL
		**N7** est-elle cotée **OUI** ?			ANOREXIE MENTALE binge eating/purging type
**O**	O1	a			si non, fin
		b			si non, fin
	O2				si non, fin
	O3	a-b-c-d-e-f			
		y a-t-il au moins **3 oui en O3 ?**			ANXIETE GENERALISEE ACTUEL

n° observation sphinx |__| |__| |__| |__|

Date de l'entretien ....

Fait par : ....

Traitement prescrit

Ou feuille d'impression informatique

NOM DU TRAITEMENT	Dosage	conditionnement	Unité	si besoin	matin	midi	soir	coucher	Classe de molécules
									AD = antidépresseur<br>NL = neuroleptique<br>EPI = anti-épileptique<br>ANT = antalgique<br>INF = anti-inflammatoire<br>D = divers<br>AX = anxiolytiques<br>A = aucun
**EXEMPLE**									
**Effexor**	**37,5**	**LP**	**cp**						AD
**Risperdal**	**2**								NL
**Tercian**			**Gttes**						NL

Inventaire de dépression de Beck

Traduction française : P. PICHOT

Date :

Doit être rempli par le participant lui-même – à compléter sur place

Instructions : *Ce questionnaire comporte 13 séries (A à M) de quatre propositions. Pour chaque série, cochez le numéro à droite qui décrit le mieux votre état actuel*

A	Je ne me sens pas triste	☐	0	A
	Je me sens cafardeux ou triste	☐	1	
	Je me sens tout le temps cafardeux ou triste, et je n'arrive pas à en sortir	☐	2	
	Je suis si triste et si malheureux que je ne peux pas le supporter	☐	3	
B	Je ne suis pas particulièrement découragé ni pessimiste au sujet de l'avenir	☐	0	B
	J'ai un sentiment de découragement au sujet de l'avenir	☐	1	
	Pour mon avenir, je n'ai aucun motif d'espérer	☐	2	
	Je ne sens qu'il y a aucun espoir pour mon avenir, et que la situation ne peut s'améliorer	☐	3	
C	Je n'ai aucun sentiment d'échec dans ma vie	☐	0	C
	J'ai l'impression que j'ai échoué dans ma vie plus que la plupart des gens	☐	1	
	Quand je regarde ma vie passée, tout ce que j'y découvre n'est qu'échecs	☐	2	
	J'ai un sentiment d'échecs complet dans toute ma vie personnelle (dans mes relations avec mes parents, mon mari, ma femme, mes enfants)	☐	3	
D	Je ne me sens pas particulièrement insatisfait	☐	0	D
	Je ne sais pas profiter agréablement des circonstances	☐	1	
	Je ne tire plus aucune satisfaction de quoi que ce soit	☐	2	
	Je suis mécontent de tout	☐	3	
E	Je ne me sens pas coupable	☐	0	E
	Je me sens mauvais ou indigne une bonne partie du temps	☐	1	
	Je me sens coupable	☐	2	
	Je me juge très mauvais, et j'ai l'impression que je ne vaux rien	☐	3	
F	Je ne suis pas déçu par moi-même	☐	0	F
	Je suis déçu par moi-même	☐	1	
	Je me dégoûte moi-même	☐	2	
	Je me hais	☐	3	
G	Je ne pense pas à me faire du mal	☐	0	G
	Je pense que la mort me libérerait	☐	1	
	J'ai des plans précis pour me suicider	☐	2	
	Si je le pouvais, je me tuerais	☐	3	
H	Je n'ai pas perdu l'intérêt pour les autres gens	☐	0	H
	Maintenant, je m'intéresse moins aux autres gens qu'autrefois	☐	1	
	J'ai perdu tout l'intérêt que je portais aux autres gens, et j'ai peu de sentiments pour eux	☐	2	
	J'ai perdu tout intérêt pour les autres, et ils m'indiffèrent totalement	☐	3	
I	Je suis capable de me décider aussi facilement que de coutume	☐	0	I
	J'essaie de ne pas avoir à prendre de décision	☐	1	
	J'ai de grandes difficultés à prendre des décisions	☐	2	
	Je ne suis plus capable de prendre la moindre décision	☐	3	
J	Je n'ai pas le sentiment d'être plus laid qu'avant	☐	0	J
	J'ai peur de paraître vieux ou disgracieux	☐	1	
	J'ai l'impression qu'il y a un changement permanent dans mon apparence physique, qui me fait paraître disgracieux	☐	2	
	J'ai l'impression d'être laid et repoussant	☐	3	
K	Je travaille aussi facilement qu'auparavant	☐	0	K
	Il me faut aussi un effort supplémentaire pour commencer à faire quelque chose	☐	1	
	Il faut que je fasse un très grand effort pour faire quoi que ce soit	☐	2	
	Je suis incapable de faire le moindre effort	☐	3	
L	Je ne suis pas plus fatigué que d'habitude	☐	0	L
	Je suis fatigué plus facilement que d'habitude	☐	1	
	Faire quoi que ce soit me fatigue	☐	2	
	Je suis incapable de faire le moindre travail	☐	3	
M	Mon appétit est toujours aussi bon	☐	0	M
	Mon appétit n'est pas aussi bon que d'habitude	☐	1	
	Mon appétit est beaucoup moins bon maintenant	☐	2	
	Je n'ai plus du tout d'appétit	☐	3	

Merci

A remplir par le participant lui-même

OCDS	Date :

Instructions : Les questions suivantes concernent votre consommation d'alcool et votre désir de contrôler cette consommation dans les **7 derniers jours**.
Veuillez **entourer** le chiffre en face de la réponse qui s'applique le mieux à votre état.

Q1 **Lorsque vous ne buvez pas d'alcool, combien de votre temps est occupé par des idées, pensées, impulsions ou images liées à la consommation d'alcool ?**

0 ☐ A aucun moment
1 ☐ moins d'une heure par jour
2 ☐ de 1 à 3 heures par jour
3 ☐ de 4 à 8 heures par jour
4 ☐ plus de 8 heures par jour

Q2 **A quelle fréquence ces pensées surviennent-elles ?**

0 ☐ jamais
1 ☐ pas plus de 8 fois par jour
2 ☐ plus de 8 fois par jour, mais pendant la plus grande partie de la journée je n'y pense pas
3 ☐ plus de 8 fois par jour et pendant la plus grande partie de la journée
4 ☐ ces pensées sont trop nombreuses pour être comptées et il ne se passe que rarement une heure sans que plusieurs de ces idées ne surviennent

Q3 **A quel point ces idées, pensées, impulsions ou images liées à la consommation d'alcool interfèrent-elles avec votre activité sociale ou professionnelle** (ou votre fonction) **? Y a-t-il quelque chose que vous ne faites pas ou ne pouvez pas faire à cause d'elles ?** (si vous ne travaillez pas actuellement, à quel point vos capacités seraient-elles atteintes si vous travailliez ?)

0 ☐ Les pensées relatives à la consommation d'alcool n'interfèrent jamais. Je peux fonctionner normalement
1 ☐ Les pensées relatives à la consommation d'alcool interfèrent légèrement avec mes activités sociales ou professionnelles, mais mes performances globales n'en sont pas affectées
2 ☐ Les pensées relatives à la consommation d'alcool interfèrent réellement avec mes activités sociales ou professionnelles, mais je peux encore m'en arranger
3 ☐ Les pensées relatives à la consommation d'alcool affectent de façon importante mes activités sociales ou professionnelles
4 ☐ Les pensées relatives à la consommation d'alcool bloquent mes activités sociales ou professionnelles

Q4 **Quelle est l'importance de la détresse ou de la perturbation que ces idées, pensées, impulsions ou images liées à la consommation d'alcool génèrent lorsque vous ne buvez pas?**

0 ☐ Aucune
1 ☐ Légère, peu fréquente et pas trop dérangeante
2 ☐ Modérée, fréquente et dérangeante mais encore gérable
3 ☐ Sévère, très fréquente et très dérangeante
4 ☐ Extrême, presque constante et bloquant les capacités

Q5 **Lorsque vous ne buvez pas, à quel point faites-vous des efforts pour résister à ces pensées ou essayer de les repousser ou de les détourner de votre attention quand elles entrent dans votre esprit?** (Evaluez vos efforts faits pour résister à ces pensées, et non votre succès ou votre échec à les contrôler réellement)

0 ☐ Mes pensées sont si minimes que je n'ai pas besoin de faire d'effort pour y résister. Si j'ai de telles pensées, je fais toujours l'effort d'y résister
1 ☐ J'essaie d'y résister la plupart du temps
2 ☐ Je fais quelques efforts pour y résister
3 ☐ Je me laisse aller à toutes ces pensées sans essayer de les contrôler, mais je le fais avec quelque hésitation
4 ☐ Je me laisse aller complètement et volontairement à toutes ces pensées

Q6 **Lorsque vous ne buvez pas, à quel point arrivez-vous à arrêter ces pensées ou à vous en détourner ?**

0 ☐ Je réussis complètement à arrêter ou à me détourner de telles pensées
1 ☐ Je suis d'habitude capable d'arrêter ces pensées ou de me détourner d'elles avec quelques efforts et de la concentration
2 ☐ Je suis parfois capable d'arrêter de telles pensées ou de m'en détourner
3 ☐ Je n'arrive que rarement à arrêter de telles pensées et ne peux m'en détourner qu'avec difficulté
4 ☐ Je n'arrive que rarement à me détourner de telles pensées même momentanément

Q7 Combien de verres de boissons alcooliques buvez-vous par jour ?
0 ☐ Aucun
1 ☐ Moins d'un verre par jour
2 ☐ De 1 à 2 verres par jour
3 ☐ De 3 à 7 verres par jour
4 ☐ 8 verres ou plus par jour

Q8 Combien de jours par semaine buvez-vous de l'alcool ?
0 ☐ Aucun
1 ☐ Pas plus d'un jour par semaine
2 ☐ De 2 à 3 j ours par semaine
3 ☐ De 4 à 5 j ours par semaine
4 ☐ De 6 à 7 j ours par semaine

Q9 A quel point votre consommation d'alcool interfère-t-elle avec votre activité professionnelle ? Existe-t-il des choses que vous ne faites pas ou ne pouvez pas faire à cause de cette consommation ? (Si vous ne travaillez pas actuellement, à quel point vos capacités professionnelles seraient-elles affectées si vous travailliez ?)
0 ☐ Le fait de boire n'interfère jamais — je peux fonctionner normalement
1 ☐ Le fait de boire interfère légèrement avec mon activité professionnelle mais l'ensemble de mes capacités n'en est pas affecté
2 ☐ Le fait de boire interfère de manière certaine avec mon activité professionnelle, mais je peux m'en arranger
3 ☐ Le fait de boire affecte de façon importante mon activité professionnelle
4 ☐ Les problèmes d'alcool bloquent mes capacités de travail

Q10 A **quel point votre consommation d'alcool interfère-t-elle avec votre activité sociale ?** Existe-t-il des choses que vous ne faites pas ou ne pouvez pas faire à cause de cette consommation ?
0 ☐ Le fait de boire n'interfère jamais —je peux fonctionner normalement
1 ☐ Le fait de boire interfère légèrement avec mes activités sociales, mais l'ensemble de mes capacités n'est pas affecté
2 ☐ Le fait de boire interfère de manière certaine avec mes activités sociales, mais je peux encore m'en arranger
3 ☐ Le fait de boire affecte de façon importante mes activités sociales
4 ☐ Les problèmes d'alcool bloquent mes activités sociales

Q11 Si l'on vous empêchait de boire de l'alcool quand vous désirez prendre un verre, à quel point seriez-vous anxieux ou énervé ?
0 ☐ Je n'éprouverais ni anxiété ni irritation
1 ☐ Je ne deviendrais que légèrement anxieux ou irrité
2 ☐ L'anxiété ou l'irritation augmenterait mais resterait contrôlable
3 ☐ J'éprouverais une augmentation d'anxiété ou d'irritation très importante et dérangeante
4 ☐ J'éprouverais une anxiété ou une irritation très invalidante

Q12 A quel point faites-vous des efforts pour résister à la consommation de boissons alcooliques? (Evaluez uniquement vos efforts pour y résister et non votre succès ou votre échec à réellement contrôler cette consommation)
0 ☐ Ma consommation est si minime que je n'ai pas besoin d'y résister — si je bois, je fais l'effort de toujours y résister
1 ☐ J'essaie d'y résister la plupart du temps
2 ☐ Je fais quelques efforts pour y résister
3 ☐ Je me laisse aller presque à chaque fois sans essayer de contrôler ma consommation d'alcool, mais je le fais avec un peu d'hésitation
4 ☐ Je me laisse aller complètement et volontairement à la boisson

Q13 A quel point vous sentez-vous poussé à consommer des boissons alcooliques ?
0 ☐ Je ne me sens pas poussé du tout
1 ☐ Je me sens faiblement poussé à boire
2 ☐ Je me sens fortement poussé à boire
3 ☐ Je me sens très fortement poussé à boire
4 ☐ Le désir de boire est entièrement involontaire et me dépasse

Q14 Quel contrôle avez-vous sur votre consommation d'alcool ?
0 ☐ J'ai un contrôle total
1 ☐ Je suis habituellement capable d'exercer un contrôle volontaire sur elle
2 ☐ Je ne peux la contrôler qu'avec difficulté
3 ☐ Je dois boire et je ne peux attendre de boire qu'avec difficulté
4 ☐ Je suis rarement capable d'attendre de boire même momentanément

HAM-A Hamilton Anxiety Scale	Date :

Cotation : 0=rien 1=faible 2=modérée 3=forte 4=très forte

		0	1	2	3	4
1	**Etat d'inquiétude :**<br>Tracas, anticipation du pire, anticipation effrayante, irritabilité	☐	☐	☐	☐	☐
2	**Tension :**<br>Impression de tension, fatigabilité, réactions de sursaut, facilité à fondre en larmes, tremblements, impression de ne pas pouvoir tenir en place, impossibilité de se détendre.	☐	☐	☐	☐	☐
3	**Etat dépressif :**<br>Perte d'intérêt, manque de plaisir dans les loisirs, dépression, réveil matinal, agitation diurne	☐	☐	☐	☐	☐
4	**Conduite pendant l'entrevue :**<br>Oubli, tremblement des mains, front plissé, visage tendu, soupirs ou respiration courte, pâleur du visage, déglutition, rots, sursauts tendineux, mydriase, exophtalmie.	☐	☐	☐	☐	☐
5	**Peurs – Frayeurs :**<br>De l'obscurité, des étrangers, d'être seul, des animaux, de la circulation (auto), des foules.	☐	☐	☐	☐	☐
6	**Intellect :**<br>Difficulté de concentration, mémoire défaillante.	☐	☐	☐	☐	☐
7	**Insomnie :**<br>Difficulté de s'endormir, sommeil intermittent, sommeil non réparateur avec fatigue au réveil, rêves, cauchemars, terreurs nocturnes	☐	☐	☐	☐	☐
8	**Symptômes autonomes :**<br>Bouche sèche, érithrose, pâleur, tendance à transpirer, étourdissements, maux de tête pendant les périodes de tension, cheveux qui se dressent.	☐	☐	☐	☐	☐
9	**Symptômes génito-urinaires :**<br>Fréquence de la miction, urgence de la miction, aménorrhée, ménorragie, frigidité, éjaculation précoce, baisse ou perte de la lilbido.	☐	☐	☐	☐	☐
10	**Symptômes cardio-vasculaires :**<br>Tachycardie, palpitations, douleur thoracique, sensation des pulsations artérielles, impression de faiblesse (ou d'évanouissement), arythmie (extra-systolie).	☐	☐	☐	☐	☐
11	**Somatique – sensorielle :**<br>Troubles de la vision (flou), bouffées de chaleur et sueurs froides, impressions de faiblesse (physique), sensations de picotement.	☐	☐	☐	☐	☐
12	**Somatique – musculaire :**<br>Douleurs musculaires, sursauts musculaires, raideurs musculaires, secousses myocloniques, grincements des dents, voix hésitante (tremblotante), augmentation du tonus musculaire.	☐	☐	☐	☐	☐
13	**Symptômes gastro-intestinaux :**<br>Difficulté de déglutition, gaz, douleurs abdominales, sensations de brûlure, ballonnement abdominal, nausée, vomissements, borborygmes, relâchement des sphincters, perte de poids, constipation.	☐	☐	☐	☐	☐
14	**Symptômes respiratoires :**<br>Sensation d'oppression, impression d'étouffement, soupirs, dyspnée.	☐	☐	☐	☐	☐

**MADRS**<br>Montgomery Asberg Depression Rating Scale	Date :

	0	1	2	3	4	5	6
**Tristesse apparente**	☐ Pas de tristesse	☐	☐ Semble découragé	☐	☐ Paraît triste et malheureux la plupart du temps	☐	☐ Semble malheureux tout le temps
**Tristesse exprimée**	☐ Tristesse occasionnelle (circonstances)	☐	☐ Triste, se déride sans difficulté	☐	☐ Sentiment envahissant de tristesse	☐	☐ Tristesse, désespoir permanent
**Tension intérieure**	☐ Calme, tension intérieure passagère	☐	☐ Irritabilité, malaise mal défini occasionnels	☐	☐ Sentiments continuels de tension, panique intermittente	☐	☐ Effroi, angoisse, panique envahissants
**Réduction de sommeil**	☐ Dort comme d'habitude	☐	☐ Légère difficulté à s'endormir	☐	☐ Sommeil réduit d'environ 2 heures	☐	☐ Moins de 2 ou 3 heures de sommeil
**Réduction de l'appétit**	☐ Appétit normal ou augmenté	☐	☐ Appétit légèrement réduit	☐	☐ Pas d'appétit	☐	☐ Ne mange que si on le persuade
**Lassitude**	☐ Pas de difficultés	☐	☐ Difficultés à commencer des activités	☐	☐ Les activités routinières sont poursuivies avec effort	☐	☐ Grande lassitude. Nécessité d'aide
**Incapacité à ressentir**	☐ Intérêt normal pour l'entourage	☐	☐ Capacité réduite à prendre plaisir	☐	☐ Perte d'intérêt, perte de sentiments	☐	☐ Sentiment de paralysie émotionnelle
**Difficultés de concentration**	☐ Pas de difficultés	☐	☐ Difficultés occasionnelles	☐	☐ Difficultés à maintenir son attention	☐	☐ Incapacité à se concentrer
**Pensées pessimistes**	☐ Pas de pensées pessimistes	☐	☐ Idées intermittentes d'échec	☐	☐ Auto-accusation, culpabilité persistante	☐	☐ Idées délirantes de ruine, d'auto-accusations
**Idées de suicide**	☐ Jouit de la vie	☐	☐ Fatigué de la vie, idées de suicide passagères	☐	☐ Il vaudrait mieux être mort, idées de suicide courantes	☐	☐ Projets explicites de suicide
